# The Significance of the DUF283 Domain for the Activity of Human Ribonuclease Dicer

**DOI:** 10.3390/ijms22168690

**Published:** 2021-08-13

**Authors:** Agnieszka Szczepanska, Marta Wojnicka, Anna Kurzynska-Kokorniak

**Affiliations:** Department of Ribonucleoprotein Biochemistry, Institute of Bioorganic Chemistry Polish Academy of Sciences, 61-704 Poznan, Poland; aszczepanska@ibch.poznan.pl (A.S.); mwojnicka@ibch.poznan.pl (M.W.)

**Keywords:** Dicer, RNase III, nucleic acid annealers, RNA-binding proteins, RNA-RNA base pairing, RNA cleavage activity

## Abstract

Dicers are multidomain proteins, usually comprising an amino-terminal putative helicase domain, a DUF283 domain (domain of unknown function), a PAZ domain, two RNase III domains (RNase IIIa and RNase IIIb) and a dsRNA-binding domain. Dicer homologs play an important role in the biogenesis of small regulatory RNAs by cleaving single-stranded precursors adopting stem-loop structures (pre-miRNAs) and double-strand RNAs into short RNA duplexes containing functional microRNAs or small interfering RNAs, respectively. Growing evidence shows that apart from the canonical role, Dicer proteins can serve a number of other functions. For example, results of our previous studies showed that human Dicer (hDicer), presumably through its DUF283 domain, can facilitate hybridization between two complementary RNAs, thus, acting as a nucleic acid annealer. Here, to test this assumption, we prepared a hDicer deletion variant lacking the amino acid residues 625-752 corresponding to the DUF283 domain. The respective 128-amino acid fragment of hDicer was earlier demonstrated to accelerate base-pairing between two complementary RNAs in vitro. We show that the ΔDUF(625-752) hDicer variant loses the potential to facilitate RNA-RNA base pairing, which strongly proves our hypothesis about the importance of the DUF283 domain for the RNA-RNA annealing activity of hDicer. Interestingly, the in vitro biochemical characterization of the obtained deletion variant reveals that it displays different RNA cleavage properties depending on the pre-miRNA substrate.

## 1. Introduction

Dicer belongs to the ribonuclease III (RNase III) family of double stranded RNA (dsRNA)-specific endoribonucleases [1]. Dicer proteins generate short RNA duplexes containing functional microRNAs (miRNAs) or small interfering RNAs (siRNAs) [2], which are loaded into a multi-protein complex referred to as the RNA-induced silencing complex (RISC) [3]. During RISC activation, one strand of the RNA duplex (called the “passenger” strand) is released and degraded and the other strand (called the “guide” strand) remains in the complex and acts as a sequence-specific probe guiding RISC to complementary transcripts [4]. Depending on the degree of complementarity between the small RNA (miRNA or siRNA) and the targeted transcript, RISC binding results in either translational repression or mRNA cleavage and degradation [5]. The vast majority of cellular processes are controlled by miRNAs or siRNAs, e.g., developmental timing, growth control, differentiation [6], apoptosis, chromatin rearrangements [7] and even viral defense [8]. Consequently, a disruption of Dicer activity can initiate pathological processes like carcinogenesis, neurodegenerative, rheumatic or immune system disorders [1,9,10,11].

Human Dicer (hDicer) contains 1992 amino acid residues (220-kDa) and is one of the most structurally complex members of the RNase III family. It comprises an amino (N)-terminal helicase domain, a domain of unknown function (DUF283), Platform, Piwi-Argonaute-Zwille (PAZ) domain, a Connector helix, two RNase III domains (RNase IIIa and RNase IIIb) and a dsRNA-binding domain (dsRBD) [12,13,14]. Extensive studies have deciphered the roles of the Dicer domains in binding and processing of its canonical substrates, i.e., single-stranded hairpin precursors of miRNAs (pre-miRNAs) and dsRNAs. The helicase domain through selective interactions with the apical loop of pre-miRNAs can discriminate between substrates [15,16]. The DUF283 domain can bind single-stranded nucleic acids in vitro [17]; thus, it is possible that this domain may be involved in interactions with the apical loop of pre-miRNA hairpins as well [18]. The Platform and PAZ domains anchor the 5′ phosphate and 2-nt 3′ overhang of a substrate [19]. The RNase IIIa and RNase IIIb domains form a single dsRNA-cleavage center. Each of the domains cleaves one strand within the RNA duplex structure, generating products with 2-nt 3′-overhangs [14]. Finally, the carboxy (C)-terminal dsRBD is presumed to play an auxiliary role in RNA substrate binding [20].

The function of DUF283 is still not well understood. Structural studies have revealed that DUF283 adopts a fold typical for proteins that bind dsRNA [21,22]. However, in vitro studies have shown only trace dsRNA-binding activity of the DUF283 domain from *Arabidopsis* *thaliana* DCL4 [21] and have not demonstrated even weak dsRNA-binding activity for DUF283 of hDicer [17]. Instead, DUF283 was found to be responsible for interacting with Dicer’s protein partners in both mammals [23] and plants [21]. Additionally, results of our previous in vitro studies have revealed that the DUF283 domain of hDicer is capable of binding single-stranded nucleic acids and can accelerate base pairing between two complementary RNAs or DNAs [17]. Likewise, full-length hDicer can facilitate hybridization between short RNAs and their targets [17,24]. We hypothesize that the DUF283 domain is crucial for the RNA-RNA annealing activity of hDicer. Consequently, to test this assumption, here we prepared a hDicer deletion variant lacking the amino acid residues 625-752 corresponding to the DUF283 domain. We showed that the ΔDUF(625-752) hDicer variant loses the potential to facilitate hybridization between two complementary RNA molecules, which strongly supports our hypothesis about the importance of the DUF283 domain for the RNA-RNA annealing activity of hDicer. Moreover, the in vitro biochemical characterization of the obtained deletion variant demonstrated that it displays different RNA cleavage properties depending on the pre-miRNA substrate. Interestingly, the in cellulo studies did not show major differences in the cleavage activity of the ΔDUF(625-752) variant with respect to the investigated substrates, but suggested decreased cleavage potential of this variant in comparison with the wild-type protein.

## 2. Results

### 2.1. Production of the hDicer DUF283 Deletion Variant

**Figure 1:**
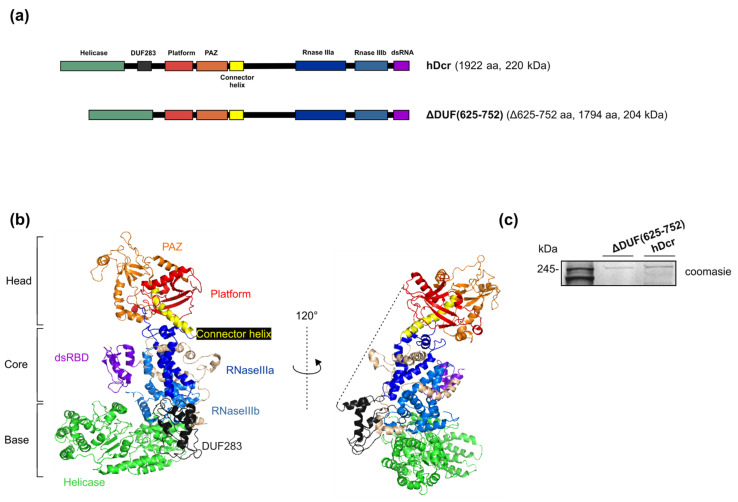
Full-length hDicer and its mutant lacking the DUF283 domain. (**a**) Schematic representation of hDicer domain architecture and the DUF283 domain deletion variant: ΔDUF(625-752). (**b**) The 3D structure of hDicer (PDB 5ZAL) visualized by PyMOL. The fragment removed in the ΔDUF(625-752) variant, i.e., DUF283 and the linker (missing in 5ZAL and drafted by the dashed line), is indicated in dark grey. (**c**) PAGE analysis of ΔDUF(625-752) and hDcr preparations. The C-terminally 3xFlag-tagged proteins were expressed in 293T NoDice cells, then they were purified by immunoprecipitation and analyzed by SDS-PAGE followed by Coomassie Blue Staining.

To investigate whether the DUF283 domain is indeed indispensable for hDicer RNA-RNA base pairing activity, we obtained the hDicer variant lacking the amino acid residues 625-752 corresponding to the DUF283 domain (a variant named ΔDUF(625-752)) (Figure 1a). This 128-amino acid fragment of hDicer was earlier demonstrated to accelerate base-pairing between two complementary RNA or DNA molecules in vitro [17]. The expression plasmid encoding the hDicer ΔDUF(625-752) variant was produced by a PCR approach using as a template the plasmid encoding the wild-type full-length hDicer fused with the 3xFlag-tag (called “hDcr”) that was earlier obtained in our laboratory [25]) (Figure 1a,b) (for details please see Materials and Methods). The expression plasmid was used for the transfection of 293T NoDice cells (human cells that do not produce hDicer) [26]. A total of 72 h after transfection, the protein was isolated and purified from cell extracts by immunoprecipitation with anti-Flag antibody conjugated to agarose beads (Figure 1c).

### 2.2. RNase Activity of the hDicer DUF283 Deletion Variant

**Figure 2:**
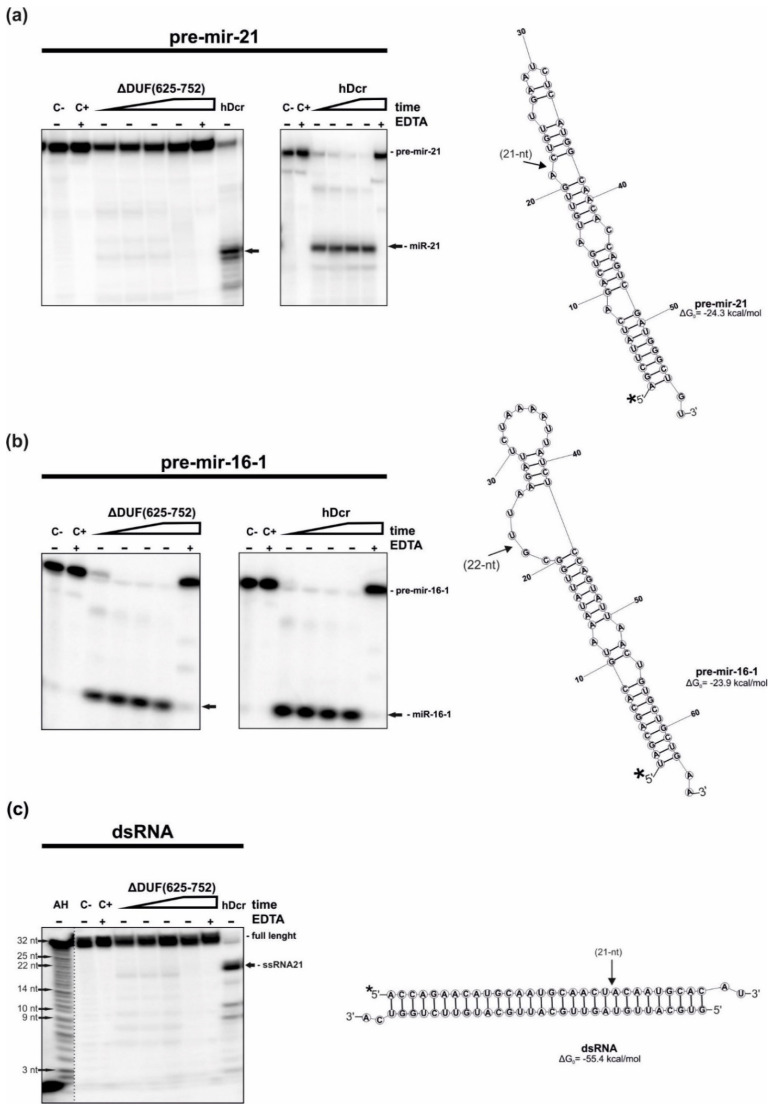
RNase activity assay of hDcr and ΔDUF(625-752). (**a**–**c**) The results of the RNA-cleavage assays involving: (**a**) pre-mir-21, (**b**) pre-mir-16-1, (**c**) 30-bp dsRNA with 2-nt 3′-overhangs. Reaction mixtures were incubated for: 10, 30, 60, 120 min (increasing time is represented by a triangle) with 18 nM of either ΔDUF(625-752) or hDcr. (C-) controls containing only the substrate in the reaction buffer. (C+) controls incubated without a protein but with 25 mM EDTA. (+EDTA) supplementation of the reaction buffer with 25 mM EDTA. (AH) Ladder generated by alkaline hydrolysis of 5′-^32^P-labeled 21-nt RNA. Dicer cleavage sites are indicated by arrows. * The asterisk indicates the ^32^P 5′-end label. The reproducible results were obtained using at least two batches of recombinant ΔDUF(625-752) and hDcr.

First, we tested the RNase activity of the ΔDUF(625-752) variant. The variant was assayed for the RNase activity using two pre-miRNA substrates: pre-mir-21 and pre-mir-16-1 and a 30-base pair (bp) RNA duplex having 2-nt 3′-overhanging ends (called “dsRNA”). The cleavage assays involved 18 nM of the protein and ~5 nM of either 5′-^32^P-labeled pre-mir-21, pre-mir-16-1 or dsRNA in which one of the two strands was 5′-^32^P-labeled. Thus, the reactions were performed under the low-turnover conditions, i.e., more than threefold molar excess of hDcr over a substrate was used. Two control reactions without the protein were also prepared: one containing only the substrate in the reaction buffer (C-) and the other containing the substrate in the reaction buffer with the addition of the Mg^2+^-chelating agent, EDTA (25 mM), (C+). As the Dicer cleavage activity is dependent on Mg^2+^, EDTA would abrogate this activity of Dicer. Thus, another control reactions included the substrate, protein and 25 mM EDTA (+EDTA). Yet another control contained the substrate and 18 nM hDcr. All reactions were carried out at 37 °C and then they were halted by adding 1 volume of 7 M UREA loading buffer and heating for 5 min at 95 °C. Reaction mixtures were separated on a 15% polyacrylamide gel with 7 M urea and 1xTBE (PAGE) and visualized by phosphorimaging (Figure 2). The cleavage assays were conducted in triplicate.

We found that in the assay with pre-mir-21, ΔDUF(625-752) generated only traces of 21-nt miRNA products (faint bands observed at the position of miR-21 generated by hDcr in the control experiment). Under the same reaction conditions, within 2 h incubation, hDcr processed almost all of the substrate (Figure 2a). In the case of the reactions carried out with the pre-mir-16-1 substrate, both ΔDUF(625-752) and hDcr produced efficiently miR-16-1. After 2 h incubation time, all pre-mir-16-1 was cut by both enzymes (Figure 2b). For the cleavage assay involving 30-bp dsRNA and ΔDUF(625-752), we noticed faint bands migrating as fast as 21-nt RNA that was generated efficiently by hDcr in the control experiment (Figure 2c). Collected data revealed that the deletion of amino acids 625-752 affects hDicer’s ability to cleave pre-mir-21 and 30-bp dsRNA, but not pre-mir-16-1, which suggests that this fragment of hDicer might be involved in substrate recognition and discrimination.

In addition, we performed the RNA cleavage assays involving the hDicer DUF283 deletion variant lacking the region spanning 630-709 amino acid residues (ΔDUF(630-709)). This variant was earlier produced and characterized by Ma E. and colleagues [27]. We found that under the same reaction conditions as those for ΔDUF(625-752), the ΔDUF(630-709) variant processed pre-mir-21, pre-mir-16-1 and 30-bp dsRNA as efficiently as the wild-type enzyme, hDcr (Figure S1).

**Figure 3:**
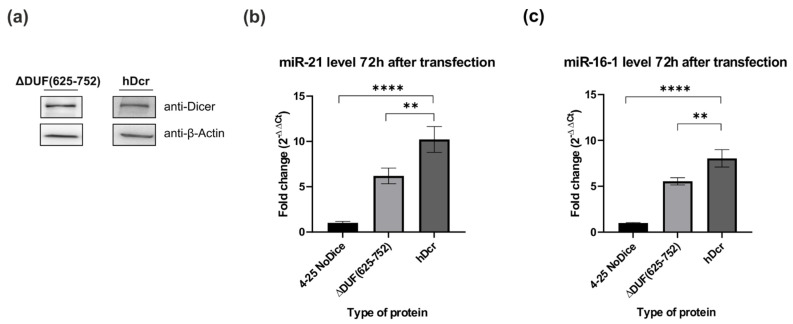
Comparison of miRNA levels produced in NoDice cells expressing ΔDUF(625-752) and hDcr. (**a**) Western blot analysis of ΔDUF(625-752) and hDcr expressed in 293T NoDice cells. Cells were harvested and analyzed by Western blotting with anti-Dicer and anti-β-Actin antibodies 72 h after transfection with the respective expression plasmids. (**b**,**c**) Results of the RT-qPCR analysis of the level of endogenous, mature miR-21 (**b**) and miR-16-1 (**c**) in the 293T NoDice cells (a negative control), or 293T NoDice cells expressing either ΔDUF(625-752) or hDcr. 72 h after transfection, cells were harvested and total RNA was isolated. miRNA levels were determined by RT-qPCR and normalized to the expression of U6 snRNA, as a reference gene. The values are averaged from three biological replicates as mean ± S.D. Data were analyzed by one-way ANNOVA test followed by Dunnett’s multiple comparisons test. The obtained *p* values are as follows, for miR-16-1: ** *p* < 0.0021, **** *p* < 0.0001; for miR-21: ** *p* < 0.0040, **** *p* < 0.0001.

The aforementioned experiments were carried out under the in vitro conditions, consequently we asked the question about the ability of the ΔDUF(625-752) variant to process pre-mir-21 and pre-mir-16-1 under in cellulo conditions. By applying the RT-qPCR approach, we assessed the relative level of miR-21-5p and miR-16-1-5p produced in 293T NoDice cells expressing the ΔDUF(625-752) variant. As a negative control, we used 293T NoDice cells treated only with a transfection reagent and as a positive control, we used 293T NoDice cells transfected with the plasmid expressing the wild-type full-length hDicer, hDcr. All cells were harvested 72 h after transfection. We found that, under similar protein expression levels (Figure 3a), both miR-21-5p and miR-16-1-5p were less abundant in 293T NoDice cells expressing ΔDUF(625-752), than in 293T NoDice cells expressing hDcr (Figure 3b,c). Precisely, a reduction in miRNA level was by about 40% (for miR-21-5p) and by about 30% (for miR-16-1-5p) compared to the respective positive control reactions. Thus, we found that miR-21-5p and miR-16-1-5p were produced with different efficiency, depending on the used system: in vitro (Figure 2a,b) or in cellulo (Figure 3b,c).

**Figure 4:**
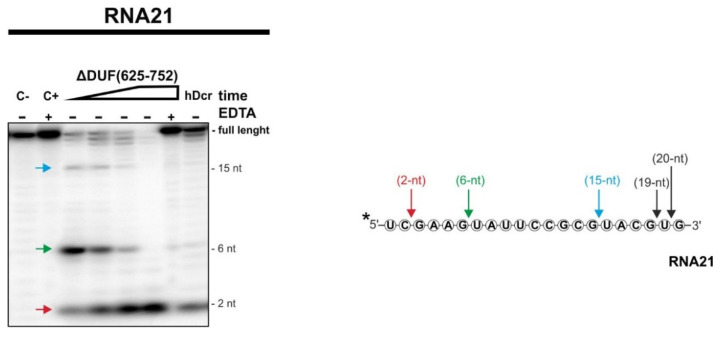
The results of the RNA-cleavage assays involving 21-nt RNA. Reaction mixtures were incubated for 10, 30, 60, 120 min for ΔDUF(625-752) (increasing time is represented by a triangle) with 18 nM of protein. (C-) control containing only the substrate in the reaction buffer. (C+) controls incubated without a protein but with 25 mM EDTA. Additional control reaction included the substrate and 18 nM hDicer protein (hDcr) incubated for 120 min. (+EDTA) supplementation of the reaction buffer with 25 mM EDTA. The asterisk indicates the ^32^P 5′-end label. The reproducible results were obtained using at least two batches of recombinant ΔDUF(625-752) and hDcr.

hDicer, apart from the canonical substrates, can bind and cut miRNA-size RNA molecules [24,25,28,29,30]; therefore, we assume that Dicer proteins might be involved in the miRNA turnover [25]. Accordingly, we performed cleavage assays with a 21-nt RNA substrate, called “RNA21”. We found that ΔDUF(625-752) generated 15-nt, 6-nt and 2-nt RNA products (Figure 4), as it was earlier observed for hDcr and the hDicer variants lacking the PAZ domain [25]. With incubation time, we observed disappearance of 15-nt and 6-nt RNA products and accumulation of 2-nt RNAs. Based on these results, we conclude that 2-nt RNAs were cut off from 15-nt and/or 6-nt RNAs. Consequently, 15-nt and 6-nt RNAs can be defined as primary cleavage products, while 2-nt RNAs, as secondary cleavage products. We also noticed that, under the adequate reaction conditions, processing of RNA21 was much more efficient in the case of ΔDUF(625-752), compared to hDcr.

### 2.3. The RNA-RNA Base Pairing Potential of the hDicer DUF283 Domain Deletion Variant

**Figure 5:**
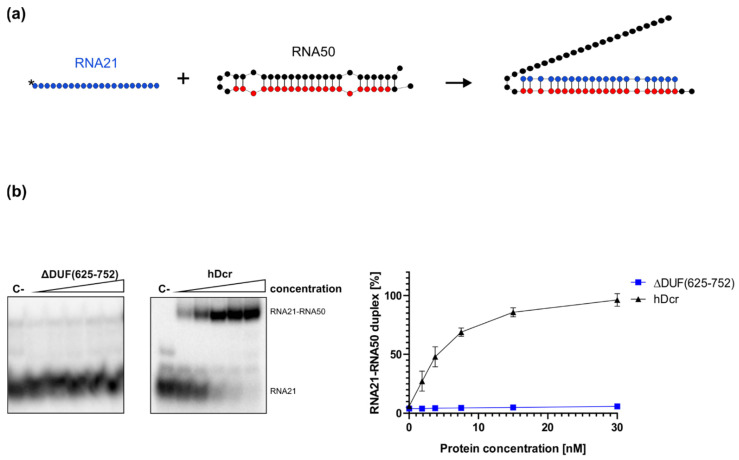
Annealing activity assay of ΔDUF(625-752) and hDcr. (**a**) Schematic representation of the experiment. RNA21 (in blue) is fully complementary to the 21-nt target sequence (in red) of RNA50. (**b**) Native PAGE gel showing the results of annealing reactions carried out for 30 min and involving 1.88, 3.75, 7.5, 15, 30 nM of either ΔDUF(625-752) or hDcr. (C-) a control incubated without the protein. The reactions were resolved in buffer containing SDS at final concentration of 0.2%. Graph presentation of the results obtained from three independent annealing assays. Error bars represent S.D. from three separate experiments.

To investigate how removing the DUF283 domain from hDicer influences its RNA-RNA base pairing activity, we carried out the annealing assay involving a pair of complementary RNAs: 21-nt RNA (RNA21) and a 50-nt RNA (called “RNA50”) adopting a hairpin structure and containing the fully complementary RNA21 target site, schematically described in Figure 5a. This pair of complementary RNAs was used in our earlier annealing assays [24]. Before the reaction, RNA50 was incubated at 95 °C for 5 min and then slowly cooled to room temperature to ensure proper folding. Then, RNA50 was mixed in annealing buffer with 5′-^32^P-labeled RNA21 at a molar ratio of approximately 1:1 and incubated for 30 min with increasing amounts of ΔDUF(625-752) (1.88, 3.75, 7.5, 15, 30 nM) at 37 °C (Figure 5b). The same set of reactions was carried out for hDcr. Spontaneous annealing was determined by excluding the enzyme in the assay mixture (control reaction (C-)). Based on the results obtained from three independent experiments, for each reaction we calculated the percentage ratios between the fraction containing the RNA21-RNA50 duplex and the free RNA21 fraction. The average percentage content of the RNA21-RNA50 duplex was plotted against the protein concentration (Figure 5b, right panel). The collected results demonstrated that, in contrast to hDcr, ΔDUF(625-752) did not support base pairing between RNA21 and RNA50 under the applied reaction conditions. Neither we observed the RNA-RNA base pairing activity for the ΔDUF(630-709) variant (Figure S2). Altogether, collected data strongly support our hypothesis that the DUF283 domain is indispensable for hDicer RNA-RNA annealing activity.

## 3. Discussion

Initially, it was suggested that the DUF283 domain is critical for pre-miRNA processing, because the in vitro cleavage activity is lost in human and *Drosophila melanogaster* Dicer variants lacking both DUF283 and the helicase domain [31,32]. However, subsequent in vitro studies demonstrated that the deletion of only DUF283 decreases the cleavage of dsRNAs by hDicer, without affecting the cleavage of pre-miRNA substrates [27]. The mentioned DUF283 deletion variant, produced by Ma E. and colleagues, lacks the region spanning 630-709 amino acid residues (the ΔDUF(630-709) variant), (Figure S1) [27]. The DUF283 deletion variant produced in our laboratory lacks the region between amino acids 625 and 752, including a linker joining the DUF283 domain to Platform (the ΔDUF(625-752) variant), (Figure 1a,b). Although removing this linker might affect the spatial organization of hDicer domains, we found that the ΔDUF(625-752) variant is successfully expressed in NoDice cells (Figure 1c). After purification, this protein retained its integrity and activity up to 12 months when stored at −20 °C. The three-dimensional structure of Dicer resembles the letter L [18,33]. Within this structure, the head, core and base can be distinguished. The head constitutes the PAZ and the Platform domains, the RNase III domains are in the core, whereas the helicase domain forms the base (Figure 1b). We can deduce that removing the linker, that joins the base to the head, precludes a proper positioning of the base (i.e., the helicase domain) in relation to the head (i.e., PAZ and Platform). Consequently, we assume that the orientation of the helicase domain differs between the ΔDUF(625-752) variant and the wild-type hDicer or the ΔDUF(630-709) variant. However, the helicase domain was shown to be dispensable for processing of both pre-miRNA and dsRNA substrates by hDicer [27]. Thus, mispositioning of the helicase domain should not abolish the cleavage activity of the ΔDUF(625-752) variant. Indeed, we found that, under the in vitro conditions, the ΔDUF(625-752) variant processed pre-mir-16-1 as efficiently as the wild-type enzyme (Figure 2b). In contrast to the case of pre-mir-16-1, the deletion of amino acids 625-752 compromised hDicer ability to cleave pre-mir-21 and 30-bp dsRNA in vitro (Figure 2a,c). What are the differences between pre-mir-16-1 and pre-mir-21 substrates? We can notice that pre-mir-16-1 contains large internal loops and bulges and has relaxed terminal loop region, whereas pre-mir-21 adopts more compact structure, with the small terminal loop (Figure 2). Therefore, taking into account the content of paired nucleotides, the structure of pre-mir-21 is more similar to the structure of the dsRNA substrate, rather than to the structure of pre-mir-16-1. Single-stranded regions of pre-mir-16-1 provide the greater flexibility of this substrate, compared to the other two substrates. Accordingly, we assume that pre-mir-16-1, due its flexibility, may better fit into the cleavage center of the ΔDUF(625-752) variant. In contrast to ΔDUF(625-752), the ΔDUF(630-709) variant processed all tested canonical substrates at least as efficiently as the wild-type enzyme (Figure S1). These data further support our hypothesis that the spatial orientation of the domains differs between ΔDUF(625-752) and ΔDUF(630-709) variants. Additionally, the collected data suggest that not only the helicase domain [15], but the DUF283 domain may as well play an important role in substrate recognition and discrimination. Importantly, as mentioned above, the removal of both the helicase and the DUF283 domains was shown to abolish in vitro processing of pre-miRNA substrates by the respectively truncated hDicer variant [31]. Collectively, based on the literature data and results of the cleavage assays generated for ΔDUF(625-752) and ΔDUF(630-709), we hypothesize that factors influencing the spatial orientation of hDicer domains may impact the enzyme’s substrate specificity and its cleavage properties.

Nevertheless, in contrast to the in vitro RNase cleavage assays (Figure 2), we found that in NoDice cells expressing ΔDUF(625-752), the levels of miR-21-5p and miR-16-1-5p were similarly decreased (by about 40% and 30%, respectively) compared to the control cell lines producing hDcr (Figure 3). These results indicate the possible involvement of cellular factors, interacting with pre-miRNAs, in the studied process.

We also conclude that the RNase III cleavage center of the ΔDUF(625-752) variant is accessible for miRNA/siRNA-size RNAs (Figure 4). The collected data showed that ΔDUF(625-752) generates the same cleavage pattern for 21-nt RNA as the wild-type enzyme and the PAZ deletion variant, which was reported in our previous studies [25]. Accordingly, taking into account the results of this and earlier studies [25], we deduce that the cleavage of miRNA-size RNAs by hDicer neither involves DUF283 nor PAZ domains. As we proposed earlier, we believe that Dicer might be involved in the miRNA turnover by promoting degradation of passenger strands that are discarded from the RISC complex [25].

Finally, we found that the deletion of the DUF283 domain (either the fragment spanning amino acids 625-752 or the fragment spanning amino acids 630-709) abolishes hDicer RNA-RNA annealing activity in vitro (Figure 5) and (Figure S2). Altogether the presented data provide further insight into the role of the DUF283 domain for the activity of human ribonuclease Dicer. The next challenge will be to translate all these results into the in vivo activity of Dicer-type proteins.

## 4. Materials and Methods

### 4.1. Oligonucleotides

DNA primers and RNA oligonucleotides were purchased from Genomed (Warsaw, Poland) and FutureSynthesis (Poznan, Poland), respectively. Sequences of all oligonucleotides used in this study are listed in Table 1.

### 4.2. ^32^P Labeling of Oligonucleotides

The 5′-end labeling of oligonucleotides was performed using 10 pmol of RNA, 1 µL γ-^32^P-ATP (Hartman Analytic GmbH, Braunschweig, Germany) and 10 U T4 polynucleotide kinase (Thermo Fisher Scientific, Waltham, MA, USA) in a final volume of 10 µL. The composition and reaction conditions were in accordance with the manufacturer’s instructions. Before preparing the reaction mixture, the RNA was denatured by heating to 95 °C and rapidly cooling on ice. The radiolabeled oligonucleotides were PAGE-purified in 8% denaturing polyacrylamide gels and resuspended in water to a final concentration of approximately 10,000 cpm/µL. The homogeneity of the material after gel purification was checked by electrophoresis in 15% PAA under denaturing conditions. Gels were exposed to a phosphorimager plate, which was subsequently scanned with FLA-5100 Fluorescent Image Analyzer (Fujifilm, Minato, Tokyo, Japan) to visualize the bands.

### 4.3. Preparation of dsRNA

To prepare a dsRNA substrate, non-labeled strand (RNA_sense) was hybridized, at a molar ratio of approximately 1:1, with ^32^P-labeled complementary strand (RNA_ov) in buffer containing 50 mM NaCl, 2.5 mM MgCl_2_ and 20 mM Tris-HCl, pH 7.5, by heating up to 95 °C and then slowly cooling down to room temperature. Next, the reaction mixtures were analyzed on 10% native polyacrylamide gels to check whether the double-stranded complexes were free of single-stranded species.

### 4.4. Preparation of the ∆DUF(625-752) and ΔDUF(630-709) Expression Plasmids

The expression plasmids encoding the ∆DUF(625-752) and ΔDUF(630-709) variants were prepared using PCR amplification. All primers were designed based on the cDNA encoding transcript variant 2 of human *DICER1* (NM_030621.4). A template in the PCR amplification was the plasmid (a derivative of SureVector expression vector (Agilent, Santa Clara, CA, USA)) encoding the full-length wild-type hDicer fused at its C-terminus with the 3xFlag peptide, that was earlier prepared in our laboratory [25]. In the reaction we used the following primers: (i) for ∆DUF(625-752), f_∆DUF(625-752) (forward) and r_∆DUF(625-752) (reverse); (ii) for ΔDUF(630-709), f_∆DUF(630-709) (forward) and r_∆DUF(630-709) (reverse). The PCR amplification involved PfuUltra II Fusion polymerase (Agilent). The obtained PCR product: a cDNA of the plasmid including the sequence of the full-length wild-type hDicer missing the fragment encoding the DUF283 domain (either the 384-nt sequence corresponding to amino acids 625-752 of hDicer or the 240-nt sequence corresponding to amino acids 630-709 of hDicer), was phosphorylated by T4 polynucleotide kinase (Thermo Fisher Scientific, Waltham, MA, USA) and ligated by T4 DNA Ligase (Thermo Fisher Scientific). The prepared constructs were sequenced by Sanger method (Genomed, Warsaw, Poland). The results of sequencing reactions are presented in Figure S3.

### 4.5. Cell Culture and Transfection

293T NoDice cells [26] were cultured in DMEM (Gibco, Thermo Fisher Scientific, Waltham, MA, USA) supplemented with 10% FBS (Gibco), Penicillin-Streptomycin (100 U/mL of penicillin and 100 μg/mL of streptomycin, Gibco) and 1 mM Sodium Pyruvate (Gibco), as described in Bogerd et al. [26]. Transfection was carried out by DharmaFECT kb DNA Transfection Reagent (Dharmacon, Lafayette, CO, USA), according to the manufacturer’s instructions. 293T NoDice cell line was kindly provided by Prof. Bryan R. Cullen.

### 4.6. Immunoprecipitation

Cells were harvested 72 h after transfection by centrifugation at 1500 rpm for 3 min and suspended in lysis buffer (30 mM Hepes pH 7.4, 100 mM KCl, 5 mM MgCl_2_, 10% glycerol, 0.5 mM DTT and 0.2% Tergitol) containing 1 × protease inhibitor mix without EDTA (Roche, Warsaw, Poland) and broken by passing through a 0.9 mm × 40 mm needle. Lysates were centrifuged at 13,000 rpm for 5 min at 4 °C. The supernatant was incubated overnight on a rotator with ANTI-FLAG^®^M2 Affinity Gel (Merck, Darmstadt, Germany) that was pre-washed with TBS buffer. After incubation, the beads were washed five times with TBS buffer. Isolated protein was eluted with 100 μg/mL 3XFlag Peptide (Sigma, Kawasaki, Japan). Purified protein was suspended in the buffer (20 mM Tris pH 7.5, 50 mM NaCl, 10% glycerol and 0.25% Triton X-100). Protein concentration was estimated by the Bradford assay (BioRad, Irvine, CA, USA), relative to a BSA standard curve and on SDS-PAGE with BSA as a standard. Proteins were concentrated using Amicon filters (Merck) in the respective buffer (as described above) enriched with 40% glycerol and stored at −20 °C.

### 4.7. Western Blot Analysis

Obtained proteins were separated on 8% SDS-PAGE and electro-transferred onto a PVDF membrane (Thermo Fisher Scientific, Waltham, MA, USA). For hDicer and hDicer deletion variant, the blots were probed with a mouse monoclonal primary anti-Dicer antibody mapping at the C-terminus of hDicer (1:300, Santa Cruz Biotechnology, Dallas, TX, USA), for β-Actin, the blots were probed with a rabbit monoclonal primary anti-β-Actin antibody (1:1100, Cell Signaling Technology, Danvers, MS, USA) and subsequently with HRP-conjugated secondary antibody, anti-mouse or anti-rabbit (1:5000, Jackson ImmunoResearch Laboratories, Inc., Cambridgeshire, UK). The immunoreactions were detected using SuperSignal^TM^West Pico PLUS Chemiluminescent Substrate (Thermo Fisher Scientific, Waltham, MA, USA).

### 4.8. The RNA Cleavage Assay

The cleavage assay was performed in 10 μL reactions containing 50 mM NaCl, 2.5 mM MgCl_2_ and 20 mM Tris-HCl, pH 7.5, 5′-^32^P-labeled substrate (10,000 cpm, approximately 5 nM) and 18 nM of the protein (hDcr or ∆DUF(625-752), or ∆DUF(630-709)). In addition, a reaction mixture without the protein was prepared as a control. In controls including EDTA, the reaction buffer was supplemented with the chelating agent to the final concentration of 25 mM. Reactions were carried out at 37 °C for 10, 30, 60 and 120 min with the addition of the commercial RNase-inhibitor cocktail (NEB, Ipswich, USA). The reactions were stopped by the addition of 1 volume of 7 M urea loading buffer and heating for 5 min at 95 °C. Samples were separated on a 15% denaturing polyacrylamide gel in 1 × TBE running buffer.

### 4.9. Reverse Transcription and Quantitative PCR

The RNA was extracted using a standard TRIzol protocol (Invitrogen, Waltham, MA, USA). The RNA quantity and quality were measured using NanoDrop 2000 (Thermo Fisher Scientific, Waltham, MA, USA). For reverse transcription (RT), 8 µg of total RNA was used. RT was performed by using Mir-X™ miRNA First-Strand Synthesis Kit (Takara Bio, Canada, USA) in accordance with the manufacturer’s instructions. RT-qPCR was performed using iTaq™ Universal SYBR^®^ Green Supermix (Bio-Rad, Irvine, CA, USA). For miRNA analysis, 8 ng of total cDNA was used. Amplification was performed using the Bio-Rad CFX96™ system (Bio-Rad) and the software determined C_t_ thresholds. The relative expression level of miRNA was evaluated by the 2^−ΔΔCt^ method [34]. miRNA levels were normalized to U6 small nuclear RNA as a reference gene. Control reactions lacking template were performed to verify clean backgrounds in all samples. The results from three independent experiments were presented as mean ± S.D. One-way ANNOVA test followed by Dunnett’s multiple comparisons test was used for statistical evaluation. Statistical analyses were performed using GraphPad Prism 6 Software (GraphPad Software, San Diego, CA, USA). The primer sequences used for qRT-PCR are listed in Table 1.

### 4.10. Annealing Assay

The reactions were carried out in 10 μL volumes. As substrates, we used the same RNA pair as in our previous annealing assays [24]. Precisely, each reaction set contained 10,000 cpm (approximately 5 nM) of the 5′-end ^32^P-labeled RNA21 and 5 nM of long complementary RNA (RNA50). The molecules were mixed in annealing buffer (50 mM NaCl, 20 mM Tris–HCl pH 7.5) and incubated for 30 min at 37 °C with dilutions of hDcr or its ∆DUF(625-752), or ΔDUF(630-709) variant (1.88, 3.75, 7.5, 15, 30 nM).

### 4.11. Gel Imaging and Analysis

The data were collected using a Fujifilm FLA-5100 Fluorescent Image Analyzer (Fujifilm, Minato, Tokyo, Japan). The amounts of ^32^P-labeled substrates and products were determined from the intensity of the respective bands in the gels measured by MultiGauge 3.0 software (Fujifilm, Minato, Tokyo, Japan). The diagrams from annealing experiments were created using GraphPad Prism 6 Software (GraphPad Software, San Diego, CA, USA). In the case of all diagrams, error bars represent S.D. values calculated based on three independent experiments.

## Figures and Tables

**Table 1:** Sequences of oligonucleotides used in the experiments.

Name	Sequence (5′ → 3′)
RNA_sense *	GUGCAUUGUAGUUGCAUUGCAUGUUCUGGUCA
**RNA_ov**	ACCAGAACAUGCAAUGCAACUACAAUGCACAU
**pre-mir-16-1**	UAGCAGCACGUAAAUAUUGGCGUUAAGAUUCUAAAAUUAUCUCCAGUAUUAACUGUGCUGCUGAA
**pre-mir-21**	AGCUUAUCAGACUGAUGUUGACUGUUGAAUCUCAUGGCAACACCAGUCGAUGGGCUGU
**RNA21**	UCGAAGUAUUCCGCGUACGUG
**RNA50**	GGUUGAACUAUUUCGUGUAUCUGGAAACACGUACGCGGAAUACUUCGAUU
**f_∆DUF**(**625-752**)	ATTCCAGAGTGTTTGAGGGATAGTTATCCCAGACCTG
**r_∆DUF**(**625-752**)	TCGTGGACCACCATCGTCAGGCCTCAACAC
**f_∆DUF**(**630-709**)	GACCATTTGATGCCAGTTGGGAAAGAGACT
**r_∆DUF**(**630-709**)	GGCCGTGTTGATTGTGACTCGTGGACCAC
**f.miR-21-5p**	GCTTATCAGACTGATGTTGAAA
**f.miR-16-1-5p**	GCACGTAAATATTGGCGAA

* RNA_sense serves as a complementary strand to RNA_ov to form dsRNA. The complementary sequences between RNA21 and RNA50 are underlined.

## Data Availability

Not applicable.

## Supplementary Materials

The following are available online at https://www.mdpi.com/article/10.3390/ijms22168690/s1.
