# Supplementary material for: The Significance of the DUF283 Domain for the Activity of Human Ribonuclease Dicer
Source: Int J Mol Sci. 2021 Aug 13;22(16):8690. doi: 10.3390/ijms22168690 (PMC8395393; doi:10.3390/ijms22168690)
Supplement: Supplementary file 1 [file ijms-22-08690-s001.zip › Figure S1.pdf]

SUPPLEMENTARY MATERIALS

The significance of the DUF283 domain for the activity of human ribonuclease Dicer

Agnieszka Szczepanska, Marta Wojnicka and Anna Kurzynska-Kokorniak *

Department of Ribonucleoprotein Biochemistry, Institute of Bioorganic Chemistry Polish Academy of Sciences, Poznan, 61-704, Poland

* Correspondence: Anna Kurzynska-Kokorniak: akurzyns@man.poznan.pl; + 48 61 852 85 03 ext. 1264.

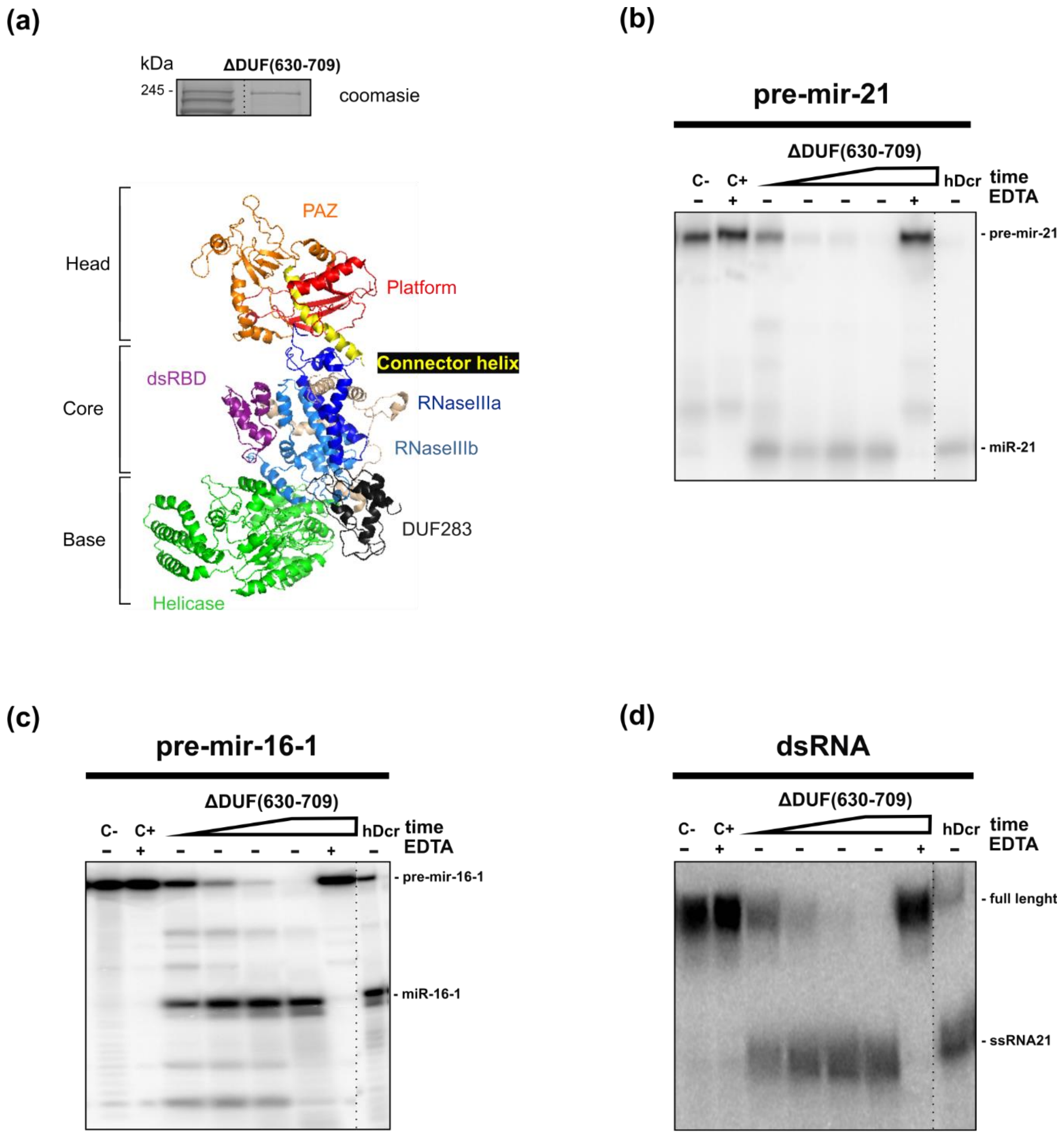

Figure S1. RNase activity assay of ΔDUF(630-709). (**a**) PAGE analysis of ΔDUF(630-709) (upper panel). The C-terminally 3xFlag-tagged protein was expressed in 293T NoDice cells, then it was purified by immunoprecipitation and analyzed by SDS-PAGE followed by Coomassie Blue Staining. The 3D structure of hDicer (PDB 5ZAL) visualized by PyMOL (bottom panel). The fragment removed in the ΔDUF(630-709) variant is indicated in dark grey. (**b-d**) The results of the RNA-cleavage assays involving: (**b**) pre-mir-21, (**c**) pre-mir-16-1, (**d**) 30-bp dsRNA with 2-nt 3´-overhangs. Reaction mixtures were incubated for 10, 30, 60, 120 min (increasing time is represented by a triangle) with 18 nM of ΔDUF(630-709). (C-) controls containing only the substrate in the reaction buffer. (C+) controls incubated without a protein but with 25 mM EDTA. Additional control reactions included the substrate and 18 nM hDicer protein (hDcr) incubated for 120 min. (+EDTA) supplementation of the reaction buffer with 25 mM EDTA. The reproducible results were obtained using at least two batches of ΔDUF(630-709).
