# Supplementary material for: The Significance of the DUF283 Domain for the Activity of Human Ribonuclease Dicer
Source: Int J Mol Sci. 2021 Aug 13;22(16):8690. doi: 10.3390/ijms22168690 (PMC8395393; doi:10.3390/ijms22168690)
Supplement: Supplementary file 1 [file ijms-22-08690-s001.zip › Figure S2.pdf]

SUPPLEMENTARY MATERIALS

The significance of the DUF283 domain for the activity of human ribonuclease Dicer

Agnieszka Szczepanska, Marta Wojnicka and Anna Kurzynska-Kokorniak *

Department of Ribonucleoprotein Biochemistry, Institute of Bioorganic Chemistry Polish Academy of Sciences, Poznan, 61-704, Poland

* Correspondence: Anna Kurzynska-Kokorniak: akurzyns@man.poznan.pl; + 48 61 852 85 03 ext. 1264.

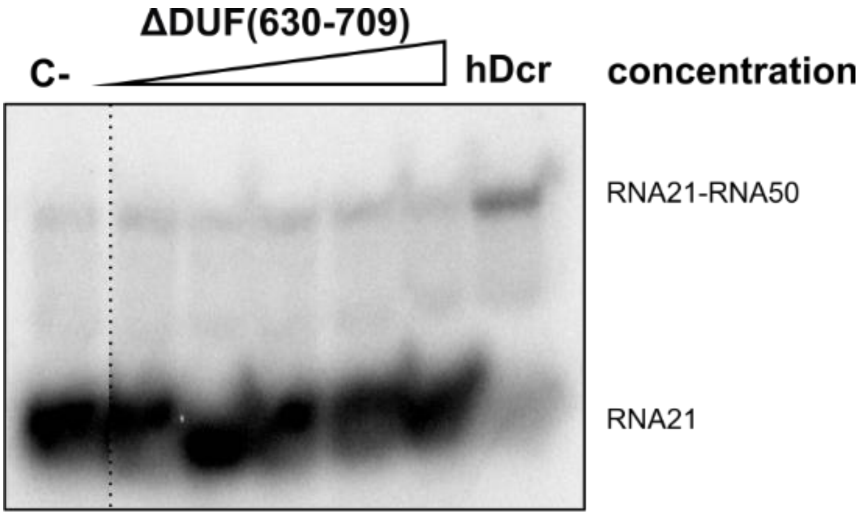

Figure S2. RNA-RNA annealing activity assay of ΔDUF(630-709). Native PAGE gel showing the results of annealing reactions carried out for 30 min and involving 1.88, 3.75, 7.5, 15, 30 nM of ΔDUF(630-709). (C-): a control incubated for 30 min without the protein. (hDcr): a control incubated for 30 min with 30 nM hDcr. The reactions were resolved in buffer containing SDS at final concentration of 0.2%.
