# Supplementary material for: The Significance of the DUF283 Domain for the Activity of Human Ribonuclease Dicer
Source: Int J Mol Sci. 2021 Aug 13;22(16):8690. doi: 10.3390/ijms22168690 (PMC8395393; doi:10.3390/ijms22168690)
Supplement: Supplementary file 1 [file ijms-22-08690-s001.zip › Figure S3.pdf]

SUPPLEMENTARY MATERIALS

The significance of the DUF283 domain for the activity of human ribonuclease Dicer

Agnieszka Szczepanska, Marta Wojnicka and Anna Kurzynska-Kokorniak *

Department of Ribonucleoprotein Biochemistry, Institute of Bioorganic Chemistry Polish Academy of Sciences, Poznan, 61-704, Poland

* Correspondence: Anna Kurzynska-Kokorniak: akurzyns@man.poznan.pl; + 48 61 852 85 03 ext. 1264.

Figure S3. Sequencing data for the ΔDUF(625-752) and ΔDUF(630-709) variants. Sanger sequence analysis of the obtained genetic constructs. All primers used for sequencing were designed based on the cDNA encoding transcript variant 2 of *DICER1* (NM_030621.4). Sequencing data from each primer is indicated as SqrP2.abi, P1.abi, P3.abi, P4.abi, P6.abi, P8.abi, P10.abi, P12.abi, respectively. Sequence data analysis was carried out using DNADynamo DNA Sequencing and Analysis Software. Note: alignment of the ΔDUF(625-752) sequence (pages:2-29), alignment of ΔDUF(630-709) sequence (pages:30-58). The reference sequence (indicated as *ref*) is the sequence of hDcr cDNA variant lacking the DUF283 domain (region comprising amino acid residues 625-752 or 630-709 of hDicer).

```
                   DraI                                      Bpu10I            StuI    PstI
ref/ΔDUF(625-752)  TACCCACCCTTGTTTAAACTTTAAGAGGAGGGCCACCATGAAAAGCCCTGCTTTGCAACCCCTCAGCATGGCAGGCCTGCAGCTCATGACCCCTGCTTCC
                                                        M  K  S  P  A  L  Q  P  L  S  M  A  G  L  Q  L  M  T  P  A  S
                                                        START

SqrP2.abi          TACCCACCCTTGTTTAAACTTTAAGAGGAGGGCCACCATGAAAAGCCCTGCTTTGCAACCCCTCAGCATGGCAGGCCTGCAGCTCATGACCCCTGCTTCC
P1.abi             --------------------------------------------------------------------------GGCCTGCAGCTCATGACCCCTGCTTCC
P3.abi             -----------------------------------------------------------------------------------------------------
P4.abi             -----------------------------------------------------------------------------------------------------
P6.abi             -----------------------------------------------------------------------------------------------------
P8.abi             -----------------------------------------------------------------------------------------------------
P10.abi            -----------------------------------------------------------------------------------------------------
P12.abi            -----------------------------------------------------------------------------------------------------
```

```
                                               NcoI
ref/ΔDUF(625-752)  TCACCAATGGGTCCTTTCTTTGGACTGCCATGGCAACAAGAAGCAATTCATGATAACATTTATACGCCAAGAAAATATCAGGTTGAACTGCTTGAAGCAG
                    S  P  M  G  P  F  F  G  L  P  W  Q  Q  E  A  I  H  D  N  I  Y  T  P  R  K  Y  Q  V  E  L  L  E  A

SqrP2.abi          TCACCAATGGGTCCTTTCTTTGGACTGCCATGGCAACAAGAAGCAATTCATGATAACATTTATACGCCAAGAAAATATCAGGTTGAACTGCTTGAAGCAG
P1.abi             TCACCAATGGGTCCTTTCTTTGGACTGCCATGGCAACAAGAAGCAATTCATGATAACATTTATACGCCAAGAAAATATCAGGTTGAACTGCTTGAAGCAG
P3.abi             -----------------------------------------------------------------------------------------------------
P4.abi             -----------------------------------------------------------------------------------------------------
P6.abi             -----------------------------------------------------------------------------------------------------
P8.abi             -----------------------------------------------------------------------------------------------------
P10.abi            -----------------------------------------------------------------------------------------------------
P12.abi            -----------------------------------------------------------------------------------------------------
```

```
                                       |DraI         |Bpu10I                         |ScaI
ref/ΔDUF(625-752) CTCTGGATCATAATACCATCGTCTGTTTAAACACTGGCTCAGGGAAGACATTTATTGCAGTACTACTCACTAAAGAGCTGTCCTATCAGATCAGGGGAGA
                  A  L  D  H  N  T  I  V  C  L  N  T  G  S  G  K  T  F  I  A  V  L  L  T  K  E  L  S  Y  Q  I  R  G  D

SqrP2.abi         CTCTGGATCATAATACCATCGTCTGTTTAAACACTGGCTCAGGGAAGACATTTATTGCAGTACTACTCACTAAAGAGCTGTCCTATCAGATCAGGGGAGA
P1.abi            CTCTGGATCATAATACCATCGTCTGTTTAAACACTGGCTCAGGGAAGACATTTATTGCAGTACTACTCACTAAAGAGCTGTCCTATCAGATCAGGGGAGA
P3.abi            ----------------------------------------------------------------------------------------------------
P4.abi            ----------------------------------------------------------------------------------------------------
P6.abi            ----------------------------------------------------------------------------------------------------
P8.abi            ----------------------------------------------------------------------------------------------------
P10.abi           ----------------------------------------------------------------------------------------------------
P12.abi           ----------------------------------------------------------------------------------------------------
```

```
                                                                                        |PvuII                  |BglII
ref/ΔDUF(625-752) CTTCAGCAGAAATGGAAAAAGGACGGTGTTCTTGGTCAACTCTGCAAACCAGGTTGCTCAACAAGTGTCAGCTGTCAGAACTCATTCAGATCTCAAGGTT
                    F  S  R  N  G  K  R  T  V  F  L  V  N  S  A  N  Q  V  A  Q  Q  V  S  A  V  R  T  H  S  D  L  K  V

SqrP2.abi         CTTCAGCAGAAATGGAAAAAGGACGGTGTTCTTGGTCAACTCTGCAAACCAGGTTGCTCAACAAGTGTCAGCTGTCAGAACTCATTCAG-----------
P1.abi            CTTCAGCAGAAATGGAAAAAGGACGGTGTTCTTGGTCAACTCTGCAAACCAGGTTGCTCAACAAGTGTCAGCTGTCAGAACTCATTCAGATCTCAAGGTT
P3.abi            ----------------------------------------------------------------------------------------------------
P4.abi            ----------------------------------------------------------------------------------------------------
P6.abi            ----------------------------------------------------------------------------------------------------
P8.abi            ----------------------------------------------------------------------------------------------------
P10.abi           ----------------------------------------------------------------------------------------------------
P12.abi           ----------------------------------------------------------------------------------------------------
```

```
                     NsiI
ref/ΔDUF(625-752) GGGGAATACTCAAACCTAGAAGTAAATGCATCTTGGACAAAAGAGAGATGGAACCAAGAGTTTACTAAGCACCAGGTTCTCATTATGACTTGCTATGTCG
                   G  E  Y  S  N  L  E  V  N  A  S  W  T  K  E  R  W  N  Q  E  F  T  K  H  Q  V  L  I  M  T  C  Y  V

SqrP2.abi         ----------------------------------------------------------------------------------------------------
P1.abi            GGGGAATACTCAAACCTAGAAGTAAATGCATCTTGGACAAAAGAGAGATGGAACCAAGAGTTTACTAAGCACCAGGTTCTCATTATGACTTGCTATGTCG
P3.abi            ----------------------------------------------------------------------------------------------------
P4.abi            ----------------------------------------------------------------------------------------------------
P6.abi            ----------------------------------------------------------------------------------------------------
P8.abi            ----------------------------------------------------------------------------------------------------
P10.abi           ----------------------------------------------------------------------------------------------------
P12.abi           ----------------------------------------------------------------------------------------------------
```

```
ref/ΔDUF(625-752) CCTTGAATGTTTTGAAAAATGGTTACTTATCACTGTCAGACATTAACCTTTTGGTGTTTGATGAGTGTCATCTTGCAATCCTAGACCACCCCTATCGAGA
                  A  L  N  V  L  K  N  G  Y  L  S  L  S  D  I  N  L  L  V  F  D  E  C  H  L  A  I  L  D  H  P  Y  R  E

SqrP2.abi         ----------------------------------------------------------------------------------------------------
P1.abi            CCTTGAATGTTTTGAAAAATGGTTACTTATCACTGTCAGACATTAACCTTTTGGTGTTTGATGAGTGTCATCTTGCAATCCTAGACCACCCCTATCGAGA
P3.abi            ----------------------------------------------------------------------------------------------------
P4.abi            ----------------------------------------------------------------------------------------------------
P6.abi            ----------------------------------------------------------------------------------------------------
P8.abi            ----------------------------------------------------------------------------------------------------
P10.abi           ----------------------------------------------------------------------------------------------------
P12.abi           ----------------------------------------------------------------------------------------------------
```

```
                                                                        DraI
ref/ΔDUF(625-752) AATTATGAAGCTCTGTGAAAATTGTCCATCATGTCCTCGCATTTTGGGACTAACTGCTTCCATTTTAAATGGGAAATGTGATCCAGAGGAATTGGAAGAA
                    I  M  K  L  C  E  N  C  P  S  C  P  R  I  L  G  L  T  A  S  I  L  N  G  K  C  D  P  E  E  L  E  E

SqrP2.abi         ----------------------------------------------------------------------------------------------------
P1.abi            AATTATGAAGCTCTGTGAAAATTGTCCATCATGTCCTCGCATTTTGGGACTAACTGCTTCCATTTTAAATGGGAAATGTGATCCAGAGGAATTGGAAGAA
P3.abi            ----------------------------------------------------------------------------------------------------
P4.abi            ----------------------------------------------------------------------------------------------------
P6.abi            ----------------------------------------------------------------------------------------------------
P8.abi            ----------------------------------------------------------------------------------------------------
P10.abi           ----------------------------------------------------------------------------------------------------
P12.abi           ----------------------------------------------------------------------------------------------------
```

```
                                         AflII
ref/ΔDUF(625-752) AAGATTCAGAAACTAGAGAAAATTCTTAAGAGTAATGCTGAAACTGCAACTGACCTGGTGGTCTTAGACAGGTATACTTCTCAGCCATGTGAGATTGTGG
                   K  I  Q  K  L  E  K  I  L  K  S  N  A  E  T  A  T  D  L  V  V  L  D  R  Y  T  S  Q  P  C  E  I  V

SqrP2.abi         ----------------------------------------------------------------------------------------------------
P1.abi            AAGATTCAGAAACTAGAGAAAATTCTTAAGAGTAATGCTGAAACTGCAACTGACCTGGTGGTCTTAGACAGGTATACTTCTCAGCCATGTGAGATTGTGG
P3.abi            ----------------------------------------------------------------------------------------------------
P4.abi            ----------------------------------------------------------------------------------------------------
P6.abi            ----------------------------------------------------------------------------------------------------
P8.abi            ----------------------------------------------------------------------------------------------------
P10.abi           ----------------------------------------------------------------------------------------------------
P12.abi           ----------------------------------------------------------------------------------------------------
```

```
ref/ΔDUF(625-752) TGGATTGTGGACCATTTACTGACAGAAGTGGGCTTTATGAAAGACTGCTGATGGAATTAGAAGAAGCACTTAATTTTATCAATGATTGTAATATATCTGT
                  V  D  C  G  P  F  T  D  R  S  G  L  Y  E  R  L  L  M  E  L  E  E  A  L  N  F  I  N  D  C  N  I  S  V

SqrP2.abi         ----------------------------------------------------------------------------------------------------
P1.abi            TGGATTGTGGACCATTTACTGACAGAAGTGGGCTTTATGAAAGACTGCTGATGGAATTAGAAGAAGCACTTAATTTTATCAATGATTGTAATATATCTGT
P3.abi            ----------------------------------------------------------------------------------------------------
P4.abi            ----------------------------------------------------------------------------------------------------
P6.abi            ----------------------------------------------------------------------------------------------------
P8.abi            ----------------------------------------------------------------------------------------------------
P10.abi           ----------------------------------------------------------------------------------------------------
P12.abi           ----------------------------------------------------------------------------------------------------
```

```
ref/ΔDUF(625-752) ACATTCAAAAGAAAGAGATTCTACTTTAATTTCGAAACAGATACTATCAGACTGTCGTGCCGTATTGGTAGTTCTGGGACCCTGGTGTGCAGATAAAGTA
                   H  S  K  E  R  D  S  T  L  I  S  K  Q  I  L  S  D  C  R  A  V  L  V  V  L  G  P  W  C  A  D  K  V

SqrP2.abi         ----------------------------------------------------------------------------------------------------
P1.abi            ACATTCAAAAGAAAGAGATTCTACTTTAATTTCGAAACAGATACTATCAGACTGTCGTGCCGTATTGGTAGTTCTGGGACCCTGGTGTGCAGATAAAGTA
P3.abi            --------------------------------------------------------------------------------------------------TA
P4.abi            ----------------------------------------------------------------------------------------------------
P6.abi            ----------------------------------------------------------------------------------------------------
P8.abi            ----------------------------------------------------------------------------------------------------
P10.abi           ----------------------------------------------------------------------------------------------------
P12.abi           ----------------------------------------------------------------------------------------------------
```

```
ref/ΔDUF(625-752) GCTGGAATGATGGTAAGAGAACTACAGAAATACATCAAACATGAGCAAGAGGAGCTGCACAGGAAATTTTTATTGTTTACAGACACTTTCCTAAGGAAAA
                   A  G  M  M  V  R  E  L  Q  K  Y  I  K  H  E  Q  E  E  L  H  R  K  F  L  L  F  T  D  T  F  L  R  K

SqrP2.abi         ----------------------------------------------------------------------------------------------------
P1.abi            GCTGGAATGATGGTAAGAGAACTACAGAAATACATCAAACATGAGCAAGAGGAGCTGCACAGGAAATTTTTATTGTTTACAGACACTTTCCTAAGGAAAA
P3.abi            GCTGGAATGATGGTAAGAGAACTACAGAAATACATCAAACATGAGCAAGAGGAGCTGCACAGGAAATTTTTATTGTTTACAGACACTTTCCTAAGGAAAA
P4.abi            ----------------------------------------------------------------------------------------------------
P6.abi            ----------------------------------------------------------------------------------------------------
P8.abi            ----------------------------------------------------------------------------------------------------
P10.abi           ----------------------------------------------------------------------------------------------------
P12.abi           ----------------------------------------------------------------------------------------------------
```

```
                              DraIII
ref/ΔDUF(625-752) TACATGCACTATGTGAAGAGCACTTCTCACCTGCCTCACTTGACCTGAAATTTGTAACTCCTAAAGTAATCAAACTGCTCGAAATCTTACGCAAATATAA
                  I  H  A  L  C  E  E  H  F  S  P  A  S  L  D  L  K  F  V  T  P  K  V  I  K  L  L  E  I  L  R  K  Y  K

SqrP2.abi         ----------------------------------------------------------------------------------------------------
P1.abi            TACATGCACTATGTGAAGAGCACTTCTCACCTGCCTCACTTGACCTGAAATTTGTAACTCCTAAAGTAATCAAACTGCTCGAAATCTTACGCAAATATAA
P3.abi            TACATGCACTATGTGAAGAGCACTTCTCACCTGCCTCACTTGACCTGAAATTTGTAACTCCTAAAGTAATCAAACTGCTCGAAATCTTACGCAAATATAA
P4.abi            ----------------------------------------------------------------------------------------------------
P6.abi            ----------------------------------------------------------------------------------------------------
P8.abi            ----------------------------------------------------------------------------------------------------
P10.abi           ----------------------------------------------------------------------------------------------------
P12.abi           ----------------------------------------------------------------------------------------------------
```

```
                   NdeI
ref/ΔDUF(625-752)  ACCATATGAGCGACAGCAGTTTGAAAGCGTTGAGTGGTATAATAATAGAAATCAGGATAATTATGTGTCATGGAGTGATTCTGAGGATGATGATGAGGAT
                    P  Y  E  R  Q  Q  F  E  S  V  E  W  Y  N  N  R  N  Q  D  N  Y  V  S  W  S  D  S  E  D  D  D  E  D

SqrP2.abi          ----------------------------------------------------------------------------------------------------
P1.abi             ACCATATGAGCGACAGCAGTTTGAAAGCGTTGAGTGGTATAATAATAGAAATCAGGATAATTATGTGTCATGGAGTGATTCTGAGGATGATGATGAGGAT
P3.abi             ACCATATGAGCGACAGCAGTTTGAAAGCGTTGAGTGGTATAATAATAGAAATCAGGATAATTATGTGTCATGGAGTGATTCTGAGGATGATGATGAGGAT
P4.abi             ----------------------------------------------------------------------------------------------------
P6.abi             ----------------------------------------------------------------------------------------------------
P8.abi             ----------------------------------------------------------------------------------------------------
P10.abi            ----------------------------------------------------------------------------------------------------
P12.abi            ----------------------------------------------------------------------------------------------------
```

```
ref/ΔDUF(625-752)  GAAGAAATTGAAGAAAAAGAGAAGCCAGAGACAAATTTTCCTTCTCCTTTTACCAACATTTTGTGCGGAATTATTTTTGTGGAAAGAAGATACACAGCAG
                    E  E  I  E  E  K  E  K  P  E  T  N  F  P  S  P  F  T  N  I  L  C  G  I  I  F  V  E  R  R  Y  T  A

SqrP2.abi          ----------------------------------------------------------------------------------------------------
P1.abi             GAAGAA----------------------------------------------------------------------------------------------
P3.abi             GAAGAAATTGAAGAAAAAGAGAAGCCAGAGACAAATTTTCCTTCTCCTTTTACCAACATTTTGTGCGGAATTATTTTTGTGGAAAGAAGATACACAGCAG
P4.abi             ----------------------------------------------------------------------------------------------------
P6.abi             ----------------------------------------------------------------------------------------------------
P8.abi             ----------------------------------------------------------------------------------------------------
P10.abi            ----------------------------------------------------------------------------------------------------
P12.abi            ----------------------------------------------------------------------------------------------------
```

```
ref/ΔDUF(625-752) TTGTCTTAAACAGATTGATAAAGGAAGCTGGCAAACAAGATCCAGAGCTGGCTTATATCAGTAGCAATTTCATAACTGGACATGGCATTGGGAAGAATCA
                  V  V  L  N  R  L  I  K  E  A  G  K  Q  D  P  E  L  A  Y  I  S  S  N  F  I  T  G  H  G  I  G  K  N  Q

SqrP2.abi         ----------------------------------------------------------------------------------------------------
P1.abi            ----------------------------------------------------------------------------------------------------
P3.abi            TTGTCTTAAACAGATTGATAAAGGAAGCTGGCAAACAAGATCCAGAGCTGGCTTATATCAGTAGCAATTTCATAACTGGACATGGCATTGGGAAGAATCA
P4.abi            -----------------------------------CAAGATCCAGAGCTGGCTTATATCAGTAGCAATTTCATAACTGGACATGGCATTGGGAAGAATCA
P6.abi            ----------------------------------------------------------------------------------------------------
P8.abi            ----------------------------------------------------------------------------------------------------
P10.abi           ----------------------------------------------------------------------------------------------------
P12.abi           ----------------------------------------------------------------------------------------------------
```

```
                                                    |EcoRI
ref/ΔDUF(625-752) GCCTCGCAACAAACAGATGGAAGCAGAATTCAGAAAACAGGAAGAGGTACTTAGGAAATTTCGAGCACATGAGACCAACCTGCTTATTGCAACAAGTATT
                    P  R  N  K  Q  M  E  A  E  F  R  K  Q  E  E  V  L  R  K  F  R  A  H  E  T  N  L  L  I  A  T  S  I

SqrP2.abi         ----------------------------------------------------------------------------------------------------
P1.abi            ----------------------------------------------------------------------------------------------------
P3.abi            GCCTCGCAACAAACAGATGGAAGCAGAATTCAGAAAACAGGAAGAGGTACTTAGGAAATTTCGAGCACATGAGACCAACCTGCTTATTGCAACAAGTATT
P4.abi            GCCTCGCAACAAACAGATGGAAGCAGAATTCAGAAAACAGGAAGAGGTACTTAGGAAATTTCGAGCACATGAGACCAACCTGCTTATTGCAACAAGTATT
P6.abi            ----------------------------------------------------------------------------------------------------
P8.abi            ----------------------------------------------------------------------------------------------------
P10.abi           ----------------------------------------------------------------------------------------------------
P12.abi           ----------------------------------------------------------------------------------------------------
```

```
                                                                                   ClaI*
ref/ΔDUF(625-752) GTAGAAGAGGGTGTTGATATACCAAAATGCAACTTGGTGGTTCGTTTTGATTTGCCCACAGAATATCGATCCTATGTTCAATCTAAAGGAAGAGCAAGGG
                   V  E  E  G  V  D  I  P  K  C  N  L  V  V  R  F  D  L  P  T  E  Y  R  S  Y  V  Q  S  K  G  R  A  R

SqrP2.abi         ----------------------------------------------------------------------------------------------------
P1.abi            ----------------------------------------------------------------------------------------------------
P3.abi            GTAGAAGAGGGTGTTGATATACCAAAATGCAACTTGGTGGTTCGTTTTGATTTGCCCACAGAATATCGATCCTATGTTCAATCTAAAGGAAGAGCAAGGG
P4.abi            GTAGAAGAGGGTGTTGATATACCAAAATGCAACTTGGTGGTTCGTTTTGATTTGCCCACAGAATATCGATCCTATGTTCAATCTAAAGGAAGAGCAAGGG
P6.abi            ----------------------------------------------------------------------------------------------------
P8.abi            ----------------------------------------------------------------------------------------------------
P10.abi           ----------------------------------------------------------------------------------------------------
P12.abi           ----------------------------------------------------------------------------------------------------
```

```
                                                                                                BglII
ref/ΔDUF(625-752) CACCCATCTCTAATTATATAATGTTAGCGGATACAGACAAAATAAAAAGTTTTGAAGAAGACCTTAAAACCTACAAAGCTATTGAAAAGATCTTGAGAAA
                  A  P  I  S  N  Y  I  M  L  A  D  T  D  K  I  K  S  F  E  E  D  L  K  T  Y  K  A  I  E  K  I  L  R  N

SqrP2.abi         ----------------------------------------------------------------------------------------------------
P1.abi            ----------------------------------------------------------------------------------------------------
P3.abi            CACCCATCTCTAATTATATAATGTTAGCGGATACAGACAAAATAAAAAGTTTTGAAGAAGACCTTAAAACCTACAAAGCTATTGAAAAGATCTTGAGAAA
P4.abi            CACCCATCTCTAATTATATAATGTTAGCGGATACAGACAAAATAAAAAGTTTTGAAGAAGACCTTAAAACCTACAAAGCTATTGAAAAGATCTTGAGAAA
P6.abi            ----------------------------------------------------------------------------------------------------
P8.abi            ----------------------------------------------------------------------------------------------------
P10.abi           ----------------------------------------------------------------------------------------------------
P12.abi           ----------------------------------------------------------------------------------------------------
```

```
                                                                                                  NdeI           StuI
ref/ΔDUF(625-752) CAAGTGTTCCAAGTCGGTTGATACTGGTGAGACTGACATTGATCCTGTCATGGATGATGATGACGTTTTCCCACCATATGTGTTGAGGCCTGACGATGGT
                    K  C  S  K  S  V  D  T  G  E  T  D  I  D  P  V  M  D  D  D  D  V  F  P  P  Y  V  L  R  P  D  D  G

SqrP2.abi         ----------------------------------------------------------------------------------------------------
P1.abi            ----------------------------------------------------------------------------------------------------
P3.abi            CAAGTGTTCCAAGTCGGTTGATACTGGTGAGACTGACATTGATCCTGTCATGGATGATGATGACGTTTTCCCACCATATGTGTTGAGGCCTGACGATGGT
P4.abi            CAAGTGTTCCAAGTCGGTTGATACTGGTGAGACTGACATTGATCCTGTCATGGATGATGATGACGTTTTCCCACCATATGTGTTGAGGCCTGACGATGGT
P6.abi            ----------------------------------------------------------------------------------------------------
P8.abi            ----------------------------------------------------------------------------------------------------
P10.abi           ----------------------------------------------------------------------------------------------------
P12.abi           ----------------------------------------------------------------------------------------------------
```

```
                          EcoRI                                  BclI*
ref/ΔDUF(625-752) GGTCCACGAATTCCAGAGTGTTTGAGGGATAGTTATCCCAGACCTGATCAGCCCTGTTACCTGTATGTGATAGGAATGGTTTTAACTACACCTTTACCTG
                   G  P  R  I  P  E  C  L  R  D  S  Y  P  R  P  D  Q  P  C  Y  L  Y  V  I  G  M  V  L  T  T  P  L  P

SqrP2.abi         ----------------------------------------------------------------------------------------------------
P1.abi            ----------------------------------------------------------------------------------------------------
P3.abi            GGTCCACGAATTCCAGAGTGTTTGAGGGATAGTTATCCCAGACCTGATCAGCCCTGTTACCTGTATGTG-------------------------------
P4.abi            GGTCCACGAATTCCAGAGTGTTTGAGGGATAGTTATCCCAGACCTGATCAGCCCTGTTACCTGTATGTGATAGGAATGGTTTTAACTACACCTTTACCTG
P6.abi            ----------------------------------------------------------------------------------------------------
P8.abi            ----------------------------------------------------------------------------------------------------
P10.abi           ----------------------------------------------------------------------------------------------------
P12.abi           ----------------------------------------------------------------------------------------------------
```

```
ref/ΔDUF(625-752) ATGAACTCAACTTTAGAAGGCGGAAGCTCTATCCTCCTGAAGATACCACAAGATGCTTTGGAATACTGACGGCCAAACCCATACCTCAGATTCCACACTT
                  D  E  L  N  F  R  R  R  K  L  Y  P  P  E  D  T  T  R  C  F  G  I  L  T  A  K  P  I  P  Q  I  P  H  F

SqrP2.abi         ----------------------------------------------------------------------------------------------------
P1.abi            ----------------------------------------------------------------------------------------------------
P3.abi            ----------------------------------------------------------------------------------------------------
P4.abi            ATGAACTCAACTTTAGAAGGCGGAAGCTCTATCCTCCTGAAGATACCACAAGATGCTTTGGAATACTGACGGCCAAACCCATACCTCAGATTCCACACTT
P6.abi            ---------------------------------------------------------------TACTGACGGCCAAACCCATACCTCAGATTCCACACTT
P8.abi            ----------------------------------------------------------------------------------------------------
P10.abi           ----------------------------------------------------------------------------------------------------
P12.abi           ----------------------------------------------------------------------------------------------------
```

```
                                                  BstEII
ref/ΔDUF(625-752) TCCTGTGTACACACGCTCTGGAGAGGTTACCATATCCATTGAGTTGAAGAAGTCTGGTTTCATGTTGTCTCTACAAATGCTTGAGTTGATTACAAGACTT
                   P  V  Y  T  R  S  G  E  V  T  I  S  I  E  L  K  K  S  G  F  M  L  S  L  Q  M  L  E  L  I  T  R  L

SqrP2.abi         ----------------------------------------------------------------------------------------------------
P1.abi            ----------------------------------------------------------------------------------------------------
P3.abi            ----------------------------------------------------------------------------------------------------
P4.abi            TCCTGTGTACACACGCTCTGGAGAGGTTACCATATCCATTGAGTTGAAGAAGTCTGGTTTCATGTTGTCTCTACAAATGCTTGAGTTGATTACAAGACTT
P6.abi            TCCTGTGTACACACGCTCTGGAGAGGTTACCATATCCATTGAGTTGAAGAAGTCTGGTTTCATGTTGTCTCTACAAATGCTTGAGTTGATTACAAGACTT
P8.abi            ----------------------------------------------------------------------------------------------------
P10.abi           ----------------------------------------------------------------------------------------------------
P12.abi           ----------------------------------------------------------------------------------------------------
```

```
                                                                                          |DraI
ref/ΔDUF(625-752) CACCAGTATATATTCTCACATATTCTTCGGCTTGAAAAACCTGCACTAGAATTTAAACCTACAGACGCTGATTCAGCATACTGTGTTCTACCTCTTAATG
                   H  Q  Y  I  F  S  H  I  L  R  L  E  K  P  A  L  E  F  K  P  T  D  A  D  S  A  Y  C  V  L  P  L  N

SqrP2.abi         ----------------------------------------------------------------------------------------------------
P1.abi            ----------------------------------------------------------------------------------------------------
P3.abi            ----------------------------------------------------------------------------------------------------
P4.abi            CACCAGTATATATTCTCACATATTCTTCGGCTTGAAAAACCTGCACTAGAATTTAAACCTACAGACGCTGATTCAGCATACTGTGTTCTACCTCTTAATG
P6.abi            CACCAGTATATATTCTCACATATTCTTCGGCTTGAAAAACCTGCACTAGAATTTAAACCTACAGACGCTGATTCAGCATACTGTGTTCTACCTCTTAATG
P8.abi            ----------------------------------------------------------------------------------------------------
P10.abi           ----------------------------------------------------------------------------------------------------
P12.abi           ----------------------------------------------------------------------------------------------------
```

```
                                      |BstXI          |DraI                                        |BsmI
ref/ΔDUF(625-752) TTGTTAATGACTCCAGCACTTTGGATATTGACTTTAAATTCATGGAAGATATTGAGAAGTCTGAAGCTCGCATAGGCATTCCCAGTACAAAGTATACAAA
                  V  V  N  D  S  S  T  L  D  I  D  F  K  F  M  E  D  I  E  K  S  E  A  R  I  G  I  P  S  T  K  Y  T  K

SqrP2.abi         ----------------------------------------------------------------------------------------------------
P1.abi            ----------------------------------------------------------------------------------------------------
P3.abi            ----------------------------------------------------------------------------------------------------
P4.abi            TTGTTAATGACTCCAGCACTTTGGATATTGACTTTAAATTCATGGAAGATATTGAGAAGTCTGAAGCTCGCATAGGCATTCCCAGTACAAAGTATACAAA
P6.abi            TTGTTAATGACTCCAGCACTTTGGATATTGACTTTAAATTCATGGAAGATATTGAGAAGTCTGAAGCTCGCATAGGCATTCCCAGTACAAAGTATACAAA
P8.abi            ----------------------------------------------------------------------------------------------------
P10.abi           ----------------------------------------------------------------------------------------------------
P12.abi           ----------------------------------------------------------------------------------------------------
```

```
                                    |DraI                                           |EcoRV        |BclI*         |ClaI
ref/ΔDUF(625-752) AGAAACACCCTTTGTTTTTAAATTAGAAGATTACCAAGATGCCGTTATCATTCCAAGATATCGCAATTTTGATCAGCCTCATCGATTTTATGTAGCTGAT
                    E  T  P  F  V  F  K  L  E  D  Y  Q  D  A  V  I  I  P  R  Y  R  N  F  D  Q  P  H  R  F  Y  V  A  D

SqrP2.abi         ----------------------------------------------------------------------------------------------------
P1.abi            ----------------------------------------------------------------------------------------------------
P3.abi            ----------------------------------------------------------------------------------------------------
P4.abi            AGAAACACCCTTTGTTTTTAAATTAGAAGATTACCAAGATGCCGTTATCATTCCAAGATATCGCAATTTTGATCAGCCTCATCGATTTTATGTAGCTGAT
P6.abi            AGAAACACCCTTTGTTTTTAAATTAGAAGATTACCAAGATGCCGTTATCATTCCAAGATATCGCAATTTTGATCAGCCTCATCGATTTTATGTAGCTGAT
P8.abi            ----------------------------------------------------------------------------------------------------
P10.abi           ----------------------------------------------------------------------------------------------------
P12.abi           ----------------------------------------------------------------------------------------------------
```

```
                                                                              |SspI
ref/ΔDUF(625-752) GTGTACACTGATCTTACCCCACTCAGTAAATTTCCTTCCCCTGAGTATGAAACTTTTGCAGAATATTATAAAACAAAGTACAACCTTGACCTAACCAATC
                   V  Y  T  D  L  T  P  L  S  K  F  P  S  P  E  Y  E  T  F  A  E  Y  Y  K  T  K  Y  N  L  D  L  T  N

SqrP2.abi         ----------------------------------------------------------------------------------------------------
P1.abi            ----------------------------------------------------------------------------------------------------
P3.abi            ----------------------------------------------------------------------------------------------------
P4.abi            GTGTACACTGATCTTACCCCACTCAGTAAATTTCCTTCCCCTGAGTATGAAACTTTTGCAGAATATTATAAAACAAAGTACAACCTTGACCTAACCAATC
P6.abi            GTGTACACTGATCTTACCCCACTCAGTAAATTTCCTTCCCCTGAGTATGAAACTTTTGCAGAATATTATAAAACAAAGTACAACCTTGACCTAACCAATC
P8.abi            ----------------------------------------------------------------------------------------------------
P10.abi           ----------------------------------------------------------------------------------------------------
P12.abi           ----------------------------------------------------------------------------------------------------
```

```
                                                                                                  Eco47III
ref/ΔDUF(625-752) TCAACCAGCCACTGCTGGATGTGGACCACACATCTTCAAGACTTAATCTTTTGACACCTCGACATTTGAATCAGAAGGGGAAAGCGCTTCCTTTAAGCAG
                  L  N  Q  P  L  L  D  V  D  H  T  S  S  R  L  N  L  L  T  P  R  H  L  N  Q  K  G  K  A  L  P  L  S  S

SqrP2.abi         ----------------------------------------------------------------------------------------------------
P1.abi            ----------------------------------------------------------------------------------------------------
P3.abi            ----------------------------------------------------------------------------------------------------
P4.abi            TCAACCAGCCACTGCTGGATGTGGACCACACATCTTCAAGACTTAATCTTTTGACACCTCGACATTTGAATCAGAAGGGGAAAGCGCTTCCTTTAAGCA-
P6.abi            TCAACCAGCCACTGCTGGATGTGGACCACACATCTTCAAGACTTAATCTTTTGACACCTCGACATTTGAATCAGAAGGGGAAAGCGCTTCCTTTAAGCAG
P8.abi            ----------------------------------------------------------------------------------------------------
P10.abi           ----------------------------------------------------------------------------------------------------
P12.abi           ----------------------------------------------------------------------------------------------------
```

```
                                                    PstI
ref/ΔDUF(625-752) TGCTGAGAAGAGGAAAGCCAAATGGGAAAGTCTGCAGAATAAACAGATACTGGTTCCAGAACTCTGTGCTATACATCCAATTCCAGCATCACTGTGGAGA
                    A  E  K  R  K  A  K  W  E  S  L  Q  N  K  Q  I  L  V  P  E  L  C  A  I  H  P  I  P  A  S  L  W  R

SqrP2.abi         ----------------------------------------------------------------------------------------------------
P1.abi            ----------------------------------------------------------------------------------------------------
P3.abi            ----------------------------------------------------------------------------------------------------
P4.abi            ----------------------------------------------------------------------------------------------------
P6.abi            TGCTGAGAAGAGGAAAGCCAAATGGGAAAGTCTGCAGAATAAACAGATACTGGTTCCAGAACTCTGTGCTATACATCCAATTCCAGCATCACTGTGGAGA
P8.abi            ----------------------------------------------------------------------------------------------------
P10.abi           ----------------------------------------------------------------------------------------------------
P12.abi           ----------------------------------------------------------------------------------------------------
```

PstI

```
ref/ΔDUF(625-752) AAAGCTGTTTGTCTCCCCAGCATACTTTATCGCCTTCACTGCCTTTTGACTGCAGAGGAGCTAAGAGCCCAGACTGCCAGCGATGCTGGCGTGGGAGTCA
                   K  A  V  C  L  P  S  I  L  Y  R  L  H  C  L  L  T  A  E  E  L  R  A  Q  T  A  S  D  A  G  V  G  V

SqrP2.abi         -----------------------------------------------------------------------------------------------------
P1.abi            -----------------------------------------------------------------------------------------------------
P3.abi            -----------------------------------------------------------------------------------------------------
P4.abi            -----------------------------------------------------------------------------------------------------
P6.abi            AAAGCTGTTTGTCTCCCCAGCATACTTTATCGCCTTCACTGCCTTTTGACTGCAGAGGAGCTAAGAGCCCAGACTGCCAGCGATGCTGGCGTGGGAGTCA
P8.abi            -----------------------------------------------------------------------------------------------------
P10.abi           -----------------------------------------------------------------------------------------------------
P12.abi           -----------------------------------------------------------------------------------------------------
```

F

```
ref/ΔDUF(625-752) GATCACTTCCTGCGGATTTTAGATACCCTAACTTAGACTTCGGGTGGAAAAAATCTATTGACAGCAAATCTTTCATCTCAATTTCTAACTCCTCTTCAGC
                  R  S  L  P  A  D  F  R  Y  P  N  L  D  F  G  W  K  K  S  I  D  S  K  S  F  I  S  I  S  N  S  S  S  A

SqrP2.abi         -----------------------------------------------------------------------------------------------------
P1.abi            -----------------------------------------------------------------------------------------------------
P3.abi            -----------------------------------------------------------------------------------------------------
P4.abi            -----------------------------------------------------------------------------------------------------
P6.abi            GATCACTTCCTGCGGATTTTAGATACCCTAACTTAGACTTCGGGTGGAAAAAATCTATTGACAGCAAATCTTTCATCTCAATTTCTAACTCCTCTTCAGC
P8.abi            --------------------------------------------------------------------TCTTTCATCTCAATTTCTAACTCCTCTTCAGC
P10.abi           -----------------------------------------------------------------------------------------------------
P12.abi           -----------------------------------------------------------------------------------------------------
```

```
                  ᐧvuII                              | MunI                                                   | XbaI
ref/ΔDUF(625-752) TGAAAATGATAATTACTGTAAGCACAGCACAATTGTCCCTGAAAATGCTGCACATCAAGGTGCTAATAGAACCTCCTCTCTAGAAAATCATGACCAAATG
                    E  N  D  N  Y  C  K  H  S  T  I  V  P  E  N  A  A  H  Q  G  A  N  R  T  S  S  L  E  N  H  D  Q  M

SqrP2.abi         ----------------------------------------------------------------------------------------------------
P1.abi            ----------------------------------------------------------------------------------------------------
P3.abi            ----------------------------------------------------------------------------------------------------
P4.abi            ----------------------------------------------------------------------------------------------------
P6.abi            TGAAAATGATAATTACTGTAAGCACAGCACAATTGTCCCTGAAAATGCTGCACATCAAGGTGCTAATAGAACCTCCTCTCTAGAAAATCATGACCAAATG
P8.abi            TGAAAATGATAATTACTGTAAGCACAGCACAATTGTCCCTGAAAATGCTGCACATCAAGGTGCTAATAGAACCTCCTCTCTAGAAAATCATGACCAAATG
P10.abi           ----------------------------------------------------------------------------------------------------
P12.abi           ----------------------------------------------------------------------------------------------------
```

```
                                  | AclI
                               | PstI                                                 | BglII           | VspI
ref/ΔDUF(625-752) TCTGTGAACTGCAGAACGTTGCTCAGCGAGTCCCCTGGTAAGCTCCACGTTGAAGTTTCAGCAGATCTTACAGCAATTAATGGTCTTTCTTACAATCAAA
                  S  V  N  C  R  T  L  L  S  E  S  P  G  K  L  H  V  E  V  S  A  D  L  T  A  I  N  G  L  S  Y  N  Q

SqrP2.abi         ----------------------------------------------------------------------------------------------------
P1.abi            ----------------------------------------------------------------------------------------------------
P3.abi            ----------------------------------------------------------------------------------------------------
P4.abi            ----------------------------------------------------------------------------------------------------
P6.abi            TCTGTGAACTGCAGAACGTTGCTCAGCGAGTCCCCTGGTAAGCTCCACGTTGAAGTTTCAGCAGATCTTACAGCAATTAATGGTCTTTCTTACAATCAAA
P8.abi            TCTGTGAACTGCAGAACGTTGCTCAGCGAGTCCCCTGGTAAGCTCCACGTTGAAGTTTCAGCAGATCTTACAGCAATTAATGGTCTTTCTTACAATCAAA
P10.abi           ----------------------------------------------------------------------------------------------------
P12.abi           ----------------------------------------------------------------------------------------------------
```

```
ref/ΔDUF(625-752) ATCTCGCCAATGGCAGTTATGATTTAGCTAACAGAGACTTTTGCCAAGGAAATCAGCTAAATTACTACAAGCAGGAAATACCCGTGCAACCAACTACCTC
                  N  L  A  N  G  S  Y  D  L  A  N  R  D  F  C  Q  G  N  Q  L  N  Y  Y  K  Q  E  I  P  V  Q  P  T  T  S

SqrP2.abi         ----------------------------------------------------------------------------------------------------
P1.abi            ----------------------------------------------------------------------------------------------------
P3.abi            ----------------------------------------------------------------------------------------------------
P4.abi            ----------------------------------------------------------------------------------------------------
P6.abi            ATCTCGCCAATGGCAGTTATGATTTAGCTAACAGAGACTTTTGCCAAGGAAATCAGCTAAATTACTACAAGCAGGAAATACCCGTGCAACCAACTACCTC
P8.abi            ATCTCGCCAATGGCAGTTATGATTTAGCTAACAGAGACTTTTGCCAAGGAAATCAGCTAAATTACTACAAGCAGGAAATACCCGTGCAACCAACTACCTC
P10.abi           ----------------------------------------------------------------------------------------------------
P12.abi           ----------------------------------------------------------------------------------------------------
```

```
ref/ΔDUF(625-752) ATATTCCATTCAGAATTTATACAGTTACGAGAACCAGCCCCAGCCCAGCGATGAATGTACTCTCCTGAGTAATAAATACCTTGATGGAAATGCTAACAAA
                   Y  S  I  Q  N  L  Y  S  Y  E  N  Q  P  Q  P  S  D  E  C  T  L  L  S  N  K  Y  L  D  G  N  A  N  K

SqrP2.abi         ----------------------------------------------------------------------------------------------------
P1.abi            ----------------------------------------------------------------------------------------------------
P3.abi            ----------------------------------------------------------------------------------------------------
P4.abi            ----------------------------------------------------------------------------------------------------
P6.abi            ATATTCCATTCAGAATTTAT--------------------------------------------------------------------------------
P8.abi            ATATTCCATTCAGAATTTATACAGTTACGAGAACCAGCCCCAGCCCAGCGATGAATGTACTCTCCTGAGTAATAAATACCTTGATGGAAATGCTAACAAA
P10.abi           ----------------------------------------------------------------------------------------------------
P12.abi           ----------------------------------------------------------------------------------------------------
```

```
ref/ΔDUF(625-752) TCTACCTCAGATGGAAGTCCTGTGATGGCCGTAATGCCTGGTACGACAGACACTATTCAAGTGCTCAAGGGCAGGATGGATTCTGAGCAGAGCCCTTCTA
                   S  T  S  D  G  S  P  V  M  A  V  M  P  G  T  T  D  T  I  Q  V  L  K  G  R  M  D  S  E  Q  S  P  S

SqrP2.abi         ----------------------------------------------------------------------------------------------------
P1.abi            ----------------------------------------------------------------------------------------------------
P3.abi            ----------------------------------------------------------------------------------------------------
P4.abi            ----------------------------------------------------------------------------------------------------
P6.abi            ----------------------------------------------------------------------------------------------------
P8.abi            TCTACCTCAGATGGAAGTCCTGTGATGGCCGTAATGCCTGGTACGACAGACACTATTCAAGTGCTCAAGGGCAGGATGGATTCTGAGCAGAGCCCTTCTA
P10.abi           ----------------------------------------------------------------------------------------------------
P12.abi           ----------------------------------------------------------------------------------------------------
```

```
                                                                |Tth111I
ref/ΔDUF(625-752) TTGGGTACTCCTCAAGGACTCTTGGCCCCAATCCTGGACTTATTCTTCAGGCTTTGACTCTGTCAAACGCTAGTGATGGATTTAACCTGGAGCGGCTTGA
                  I  G  Y  S  S  R  T  L  G  P  N  P  G  L  I  L  Q  A  L  T  L  S  N  A  S  D  G  F  N  L  E  R  L  E

SqrP2.abi         ----------------------------------------------------------------------------------------------------
P1.abi            ----------------------------------------------------------------------------------------------------
P3.abi            ----------------------------------------------------------------------------------------------------
P4.abi            ----------------------------------------------------------------------------------------------------
P6.abi            ----------------------------------------------------------------------------------------------------
P8.abi            TTGGGTACTCCTCAAGGACTCTTGGCCCCAATCCTGGACTTATTCTTCAGGCTTTGACTCTGTCAAACGCTAGTGATGGATTTAACCTGGAGCGGCTTGA
P10.abi           ----------------------------------------------------------------------------------------------------
P12.abi           ----------------------------------------------------------------------------------------------------
```

```
                   DraI       SphI
ref/ΔDUF(625-752)  AATGCTTGGCGACTCCTTTTTAAAGCATGCCATCACCACATATCTATTTTGCACTTACCCTGATGCGCATGAGGGCCGCCTTTCATATATGAGAAGCAAA
                    M  L  G  D  S  F  L  K  H  A  I  T  T  Y  L  F  C  T  Y  P  D  A  H  E  G  R  L  S  Y  M  R  S  K

SqrP2.abi          ----------------------------------------------------------------------------------------------------
P1.abi             ----------------------------------------------------------------------------------------------------
P3.abi             ----------------------------------------------------------------------------------------------------
P4.abi             ----------------------------------------------------------------------------------------------------
P6.abi             ----------------------------------------------------------------------------------------------------
P8.abi             AATGCTTGGCGACTCCTTTTTAAAGCATGCCATCACCACATATCTATTTTGCACTTACCCTGATGCGCATGAGGGCCGCCTTTCATATATGAGAAGCAAA
P10.abi            ----------------------------------------------------------------------------------------------------
P12.abi            ----------------------------------------------------------------------------------------------------
```

```
                                                                      BstXI          SspI
ref/ΔDUF(625-752)  AAGGTCAGCAACTGTAATCTGTATCGCCTTGGAAAAAAGAAGGGACTACCCAGCCGCATGGTGGTGTCAATATTTGATCCCCCTGTGAATTGGCTTCCTC
                    K  V  S  N  C  N  L  Y  R  L  G  K  K  K  G  L  P  S  R  M  V  V  S  I  F  D  P  P  V  N  W  L  P

SqrP2.abi          ----------------------------------------------------------------------------------------------------
P1.abi             ----------------------------------------------------------------------------------------------------
P3.abi             ----------------------------------------------------------------------------------------------------
P4.abi             ----------------------------------------------------------------------------------------------------
P6.abi             ----------------------------------------------------------------------------------------------------
P8.abi             AAGGTCAGCAACTGTAATCTGTATCGCCTTGGAAAAAAGAAGGGACTACCCAGCCGCATGGTGGTGTCAATATTTGATCCCCCTGTGAATTGGCTTCCTC
P10.abi            ----------------------------------------------------------------------------------------------------
P12.abi            ----------------------------------------------------------------------------------------------------
```

```
                                                                                                       |SphI
ref/ΔDUF(625-752) CTGGTTATGTAGTAAATCAAGACAAAAGCAACACAGATAAATGGGAAAAAGATGAAATGACAAAAGACTGCATGCTGGCGAATGGCAAACTGGATGAGGA
                  P  G  Y  V  V  N  Q  D  K  S  N  T  D  K  W  E  K  D  E  M  T  K  D  C  M  L  A  N  G  K  L  D  E  D

SqrP2.abi         ----------------------------------------------------------------------------------------------------
P1.abi            ----------------------------------------------------------------------------------------------------
P3.abi            ----------------------------------------------------------------------------------------------------
P4.abi            ----------------------------------------------------------------------------------------------------
P6.abi            ----------------------------------------------------------------------------------------------------
P8.abi            CTGGTTATGTAGTAAATCAAGACAAAAGCAACACAGATAAATGGGAAAAAGATGAAATGACAAAAGACTGCATGCTGGCGAATGGCAAACTGGATGAGGA
P10.abi           ------------------------------------------------------------------------------------------TGGATGAGGA
P12.abi           ----------------------------------------------------------------------------------------------------
```

```
                                                                                                                |BclI*
ref/ΔDUF(625-752) TTACGAGGAGGAGGATGAGGAGGAGGAGAGCCTGATGTGGAGGGCTCCGAAGGAAGAGGCTGACTATGAAGATGATTTCCTGGAGTATGATCAGGAACAT
                    Y  E  E  E  D  E  E  E  E  S  L  M  W  R  A  P  K  E  E  A  D  Y  E  D  D  F  L  E  Y  D  Q  E  H

SqrP2.abi         ----------------------------------------------------------------------------------------------------
P1.abi            ----------------------------------------------------------------------------------------------------
P3.abi            ----------------------------------------------------------------------------------------------------
P4.abi            ----------------------------------------------------------------------------------------------------
P6.abi            ----------------------------------------------------------------------------------------------------
P8.abi            TTACGAGGAGGAGGATGAGGAGGAGGAGAGCCTGATGTGGAGGGCTCCGAAGGAAGAGGCTGACTATGAAGATGATTTCCTGGAGTATGATCAGGAACAT
P10.abi           TTACGAGGAGGAGGATGAGGAGGAGGAGAGCCTGATGTGGAGGGCTCCGAAGGAAGAGGCTGACTATGAAGATGATTTCCTGGAGTATGATCAGGAACAT
P12.abi           ----------------------------------------------------------------------------------------------------
```

```
                                                                                                  NdeI
ref/ΔDUF(625-752) ATCAGATTTATAGATAATATGTTAATGGGGTCAGGAGCTTTTGTAAAGAAAATCTCTCTTTCTCCTTTTTCAACCACTGATTCTGCATATGAATGGAAAA
                   I  R  F  I  D  N  M  L  M  G  S  G  A  F  V  K  K  I  S  L  S  P  F  S  T  T  D  S  A  Y  E  W  K

SqrP2.abi         ----------------------------------------------------------------------------------------------------
P1.abi            ----------------------------------------------------------------------------------------------------
P3.abi            ----------------------------------------------------------------------------------------------------
P4.abi            ----------------------------------------------------------------------------------------------------
P6.abi            ----------------------------------------------------------------------------------------------------
P8.abi            ATCAGATTTATAGATAATATGTTAATGGGGTCAGGAGCTTTTGTAAAGAAAATCTCTCTTTCTCCTTTTTCAACCACTGATTCTGCATATGAATGGAAAA
P10.abi           ATCAGATTTATAGATAATATGTTAATGGGGTCAGGAGCTTTTGTAAAGAAAATCTCTCTTTCTCCTTTTTCAACCACTGATTCTGCATATGAATGGAAAA
P12.abi           ----------------------------------------------------------------------------------------------------
```

```
                                                                                                    BamHI
ref/ΔDUF(625-752) TGCCCAAAAAATCCTCCTTAGGTAGTATGCCATTTTCATCAGATTTTGAGGATTTTGACTACAGCTCTTGGGATGCAATGTGCTATCTGGATCCTAGCAA
                  M  P  K  K  S  S  L  G  S  M  P  F  S  S  D  F  E  D  F  D  Y  S  S  W  D  A  M  C  Y  L  D  P  S  K

SqrP2.abi         ----------------------------------------------------------------------------------------------------
P1.abi            ----------------------------------------------------------------------------------------------------
P3.abi            ----------------------------------------------------------------------------------------------------
P4.abi            ----------------------------------------------------------------------------------------------------
P6.abi            ----------------------------------------------------------------------------------------------------
P8.abi            TGCCCAAAAAATCCTCCTTAGGTAGTATGCCATTTTCATCAGATTTTGAGGATTTTGACTACAGCTCTT-------------------------------
P10.abi           TGCCCAAAAAATCCTCCTTAGGTAGTATGCCATTTTCATCAGATTTTGAGGATTTTGACTACAGCTCTTGGGATGCAATGTGCTATCTGGATCCTAGCAA
P12.abi           ----------------------------------------------------------------------------------------------------
```

```
ref/ΔDUF(625-752) AGCTGTTGAAGAAGATGACTTTGTGGTGGGGTTCTGGAATCCATCAGAAGAAAACTGTGGTGTTGACACGGGAAAGCAGTCCATTTCTTACGACTTGCAC
                   A  V  E  E  D  D  F  V  V  G  F  W  N  P  S  E  E  N  C  G  V  D  T  G  K  Q  S  I  S  Y  D  L  H

SqrP2.abi         ----------------------------------------------------------------------------------------------------
P1.abi            ----------------------------------------------------------------------------------------------------
P3.abi            ----------------------------------------------------------------------------------------------------
P4.abi            ----------------------------------------------------------------------------------------------------
P6.abi            ----------------------------------------------------------------------------------------------------
P8.abi            ----------------------------------------------------------------------------------------------------
P10.abi           AGCTGTTGAAGAAGATGACTTTGTGGTGGGGTTCTGGAATCCATCAGAAGAAAACTGTGGTGTTGACACGGGAAAGCAGTCCATTTCTTACGACTTGCAC
P12.abi           ----------------------------------------------------------------------------------------------------
```

```
                                                                                      PvuII
ref/ΔDUF(625-752) ACTGAGCAGTGTATTGCTGACAAAAGCATAGCGGACTGTGTGGAAGCCCTGCTGGGCTGCTATTTAACCAGCTGTGGGGAGAGGGCTGCTCAGCTTTTCC
                   T  E  Q  C  I  A  D  K  S  I  A  D  C  V  E  A  L  L  G  C  Y  L  T  S  C  G  E  R  A  A  Q  L  F

SqrP2.abi         ----------------------------------------------------------------------------------------------------
P1.abi            ----------------------------------------------------------------------------------------------------
P3.abi            ----------------------------------------------------------------------------------------------------
P4.abi            ----------------------------------------------------------------------------------------------------
P6.abi            ----------------------------------------------------------------------------------------------------
P8.abi            ----------------------------------------------------------------------------------------------------
P10.abi           ACTGAGCAGTGTATTGCTGACAAAAGCATAGCGGACTGTGTGGAAGCCCTGCTGGGCTGCTATTTAACCAGCTGTGGGGAGAGGGCTGCTCAGCTTTTCC
P12.abi           ----------------------------------------------------------------------------------------------------
```

```
ref/ΔDUF(625-752) TCTGTTCACTGGGGCTGAAGGTGCTCCCGGTAATTAAAAGGACTGATCGGGAAAAGGCCCTGTGCCCTACTCGGGAGAATTTCAACAGCCAACAAAAGAA
                   L  C  S  L  G  L  K  V  L  P  V  I  K  R  T  D  R  E  K  A  L  C  P  T  R  E  N  F  N  S  Q  Q  K  N

SqrP2.abi         ----------------------------------------------------------------------------------------------------
P1.abi            ----------------------------------------------------------------------------------------------------
P3.abi            ----------------------------------------------------------------------------------------------------
P4.abi            ----------------------------------------------------------------------------------------------------
P6.abi            ----------------------------------------------------------------------------------------------------
P8.abi            ----------------------------------------------------------------------------------------------------
P10.abi           TCTGTTCACTGGGGCTGAAGGTGCTCCCGGTAATTAAAAGGACTGATCGGGAAAAGGCCCTGTGCCCTACTCGGGAGAATTTCAACAGCCAACAAAAGAA
P12.abi           ----------------------------------------------------------------------------------------------------
```

```
                                                   BalI
ref/ΔDUF(625-752) CCTTTCAGTGAGCTGTGCTGCTGCTTCTGTGGCCAGTTCACGCTCTTCTGTATTGAAAGACTCGGAATATGGTTGTTTGAAGATTCCACCAAGATGTATG
                    L  S  V  S  C  A  A  A  S  V  A  S  S  R  S  S  V  L  K  D  S  E  Y  G  C  L  K  I  P  P  R  C  M

SqrP2.abi         ----------------------------------------------------------------------------------------------------
P1.abi            ----------------------------------------------------------------------------------------------------
P3.abi            ----------------------------------------------------------------------------------------------------
P4.abi            ----------------------------------------------------------------------------------------------------
P6.abi            ----------------------------------------------------------------------------------------------------
P8.abi            ----------------------------------------------------------------------------------------------------
P10.abi           CCTTTCAGTGAGCTGTGCTGCTGCTTCTGTGGCCAGTTCACGCTCTTCTGTATTGAAAGACTCGGAATATGGTTGTTTGAAGATTCCACCAAGATGTATG
P12.abi           ----------------------------------------------------------------------------------------------------
```

```
                  |BclI*
ref/ΔDUF(625-752) TTTGATCATCCAGATGCAGATAAAACACTGAATCACCTTATATCGGGGTTTGAAAATTTTGAAAAGAAAATCAACTACAGATTCAAGAATAAGGCTTACC
                   F  D  H  P  D  A  D  K  T  L  N  H  L  I  S  G  F  E  N  F  E  K  K  I  N  Y  R  F  K  N  K  A  Y

SqrP2.abi         ----------------------------------------------------------------------------------------------------
P1.abi            ----------------------------------------------------------------------------------------------------
P3.abi            ----------------------------------------------------------------------------------------------------
P4.abi            ----------------------------------------------------------------------------------------------------
P6.abi            ----------------------------------------------------------------------------------------------------
P8.abi            ----------------------------------------------------------------------------------------------------
P10.abi           TTTGATCATCCAGATGCAGATAAAACACTGAATCACCTTATATCGGGGTTTGAAAATTTTGAAAAGAAAATCAACTACAGATTCAAGAATAAGGCTTACC
P12.abi           ----------------------------------------------------------------------------------------------------
```

```
                                                                       |Eco47III|EcoRI
ref/ΔDUF(625-752) TTCTCCAGGCTTTTACACATGCCTCCTACCACTACAATACTATCACTGATTGTTACCAGCGCTTAGAATTCCTGGGAGATGCGATTTTGGACTACCTCAT
                  L  L  Q  A  F  T  H  A  S  Y  H  Y  N  T  I  T  D  C  Y  Q  R  L  E  F  L  G  D  A  I  L  D  Y  L  I

SqrP2.abi         ----------------------------------------------------------------------------------------------------
P1.abi            ----------------------------------------------------------------------------------------------------
P3.abi            ----------------------------------------------------------------------------------------------------
P4.abi            ----------------------------------------------------------------------------------------------------
P6.abi            ----------------------------------------------------------------------------------------------------
P8.abi            ----------------------------------------------------------------------------------------------------
P10.abi           TTCTCCAGGCTTTTACACATGCCTCCTACCACTACAATACTATCACTGATTGTTACCAGCGCTTAGAATTCCTGGGAGATGCGATTTTGGACTACCTCAT
P12.abi           ----------------------------------------------------------------------------------------------------
```

```
                                           SmaI
                              SacII     XmaI
ref/ΔDUF(625-752) AACCAAGCACCTTTATGAAGACCCGCGGCAGCACTCCCCGGGGGTCCTGACAGACCTGCGGTCTGCCCTGGTCAACAACACCATCTTTGCATCGCTGGCT
                    T  K  H  L  Y  E  D  P  R  Q  H  S  P  G  V  L  T  D  L  R  S  A  L  V  N  N  T  I  F  A  S  L  A

SqrP2.abi         ----------------------------------------------------------------------------------------------------
P1.abi            ----------------------------------------------------------------------------------------------------
P3.abi            ----------------------------------------------------------------------------------------------------
P4.abi            ----------------------------------------------------------------------------------------------------
P6.abi            ----------------------------------------------------------------------------------------------------
P8.abi            ----------------------------------------------------------------------------------------------------
P10.abi           AACCAAGCACCTTTATGAAGACCCGCGGCAGCACTCCCCGGGGGTCCTGACAGACCTGCGGTCTGCCCTGGTCAACAACACCATCTTTGCATCGCTGGCT
P12.abi           -------------------------------------------------------------------------ACAACACCATCTTTGCATCGCTGGCT
```

```
                                                  EcoICRI
                            ScaI            Bpu10I SacI
ref/ΔDUF(625-752) GTAAAGTACGACTACCACAAGTACTTCAAAGCTGTCTCTCCTGAGCTCTTCCATGTCATTGATGACTTTGTGCAGTTTCAGCTTGAGAAGAATGAAATGC
                   V  K  Y  D  Y  H  K  Y  F  K  A  V  S  P  E  L  F  H  V  I  D  D  F  V  Q  F  Q  L  E  K  N  E  M

SqrP2.abi         ----------------------------------------------------------------------------------------------------
P1.abi            ----------------------------------------------------------------------------------------------------
P3.abi            ----------------------------------------------------------------------------------------------------
P4.abi            ----------------------------------------------------------------------------------------------------
P6.abi            ----------------------------------------------------------------------------------------------------
P8.abi            ----------------------------------------------------------------------------------------------------
P10.abi           GTAAAGTACGACTACCACAAGTACTTCAAAGCTGTCTCTCCTGAGCTCTTCCATGTCATTGATGACTTTGTGCAGTTTCAGCTTGAGAAGAATGAAATGC
P12.abi           GTAAAGTACGACTACCACAAGTACTTCAAAGCTGTCTCTCCTGAGCTCTTCCATGTCATTGATGACTTTGTGCAGTTTCAGCTTGAGAAGAATGAAATGC
```

```
                               |Bpu10I |BglII                                         |NcoI
ref/ΔDUF(625-752) AAGGAATGGATTCTGAGCTTAGGAGATCTGAGGAGGATGAAGAGAAAGAAGAGGATATTGAAGTTCCAAAGGCCATGGGGGATATTTTTGAGTCGCTTGC
                  Q  G  M  D  S  E  L  R  R  S  E  E  D  E  E  K  E  E  D  I  E  V  P  K  A  M  G  D  I  F  E  S  L  A

SqrP2.abi         ----------------------------------------------------------------------------------------------------
P1.abi            ----------------------------------------------------------------------------------------------------
P3.abi            ----------------------------------------------------------------------------------------------------
P4.abi            ----------------------------------------------------------------------------------------------------
P6.abi            ----------------------------------------------------------------------------------------------------
P8.abi            ----------------------------------------------------------------------------------------------------
P10.abi           AAGGAATGGATTCTGAGCTTAGGAGATCTGAGGAGGATGAAGAGAAAGAAGAGGATATTGAAGTTCCAAAGGCCATGGGGGA------------------
P12.abi           AAGGAATGGATTCTGAGCTTAGGAGATCTGAGGAGGATGAAGAGAAAGAAGAGGATATTGAAGTTCCAAAGGCCATGGGGGATATTTTTGAGTCGCTTGC
```

```
                          |BstXI
ref/ΔDUF(625-752) TGGTGCCATTTACATGGATAGTGGGATGTCACTGGAGACAGTCTGGCAGGTGTACTATCCCATGATGCGGCCACTAATAGAAAAGTTTTCTGCAAATGTA
                    G  A  I  Y  M  D  S  G  M  S  L  E  T  V  W  Q  V  Y  Y  P  M  M  R  P  L  I  E  K  F  S  A  N  V

SqrP2.abi         ----------------------------------------------------------------------------------------------------
P1.abi            ----------------------------------------------------------------------------------------------------
P3.abi            ----------------------------------------------------------------------------------------------------
P4.abi            ----------------------------------------------------------------------------------------------------
P6.abi            ----------------------------------------------------------------------------------------------------
P8.abi            ----------------------------------------------------------------------------------------------------
P10.abi           ----------------------------------------------------------------------------------------------------
P12.abi           TGGTGCCATTTACATGGATAGTGGGATGTCACTGGAGACAGTCTGGCAGGTGTACTATCCCATGATGCGGCCACTAATAGAAAAGTTTTCTGCAAATGTA
```

```
ref/ΔDUF(625-752) CCCCGTTCCCCTGTGCGAGAATTGCTTGAAATGGAACCAGAAACTGCCAAATTTAGCCCGGCTGAGAGAACTTACGACGGGAAGGTCAGAGTCACTGTGG
                   P  R  S  P  V  R  E  L  L  E  M  E  P  E  T  A  K  F  S  P  A  E  R  T  Y  D  G  K  V  R  V  T  V

SqrP2.abi         ----------------------------------------------------------------------------------------------------
P1.abi            ----------------------------------------------------------------------------------------------------
P3.abi            ----------------------------------------------------------------------------------------------------
P4.abi            ----------------------------------------------------------------------------------------------------
P6.abi            ----------------------------------------------------------------------------------------------------
P8.abi            ----------------------------------------------------------------------------------------------------
P10.abi           ----------------------------------------------------------------------------------------------------
P12.abi           CCCCGTTCCCCTGTGCGAGAATTGCTTGAAATGGAACCAGAAACTGCCAAATTTAGCCCGGCTGAGAGAACTTACGACGGGAAGGTCAGAGTCACTGTGG
```

```
                                         |DraI                                    |PstI
ref/ΔDUF(625-752) AAGTAGTAGGAAAGGGGAAATTTAAAGGTGTTGGTCGAAGTTACAGGATTGCCAAATCTGCAGCAGCAAGAAGAGCCCTCCGAAGCCTCAAAGCTAATCA
                  E  V  V  G  K  G  K  F  K  G  V  G  R  S  Y  R  I  A  K  S  A  A  A  R  R  A  L  R  S  L  K  A  N  Q

SqrP2.abi         ----------------------------------------------------------------------------------------------------
P1.abi            ----------------------------------------------------------------------------------------------------
P3.abi            ----------------------------------------------------------------------------------------------------
P4.abi            ----------------------------------------------------------------------------------------------------
P6.abi            ----------------------------------------------------------------------------------------------------
P8.abi            ----------------------------------------------------------------------------------------------------
P10.abi           ----------------------------------------------------------------------------------------------------
P12.abi           AAGTAGTAGGAAAGGGGAAATTTAAAGGTGTTGGTCGAAGTTACAGGATTGCCAAATCTGCAGCAGCAAGAAGAGCCCTCCGAAGCCTCAAAGCTAATCA
```

```
ref/ΔDUF(625-752) ACCTCAGGTTCCCAATAGCGGTGGCGGAGGTTCTGGAGGCGGTGGAAGTGACTACAAGGACGACGATGACAAGGATTACAAAGACGATGATGACAAGGAC
                   P  Q  V  P  N  S  G  G  G  G  S  G  G  G  G  S  D  Y  K  D  D  D  D  K  D  Y  K  D  D  D  D  K  D

SqrP2.abi         ----------------------------------------------------------------------------------------------------
P1.abi            ----------------------------------------------------------------------------------------------------
P3.abi            ----------------------------------------------------------------------------------------------------
P4.abi            ----------------------------------------------------------------------------------------------------
P6.abi            ----------------------------------------------------------------------------------------------------
P8.abi            ----------------------------------------------------------------------------------------------------
P10.abi           ----------------------------------------------------------------------------------------------------
P12.abi           ACCTCAGGTTCCCAATAGCGGTGGCGGAGGTTCTGGAGGCGGTGGAAGTGACTACAAGGACGACGATGACAAGGATTACAAAGACGATGATGACAAGGAC
```

```
                                                             XhoI
ref/ΔDUF(625-752) TATAAGGACGATGACGATAAGTAACATTTGGTTTAGTGTACAATATCTCCTCGAG
                   Y  K  D  D  D  D  K  *  H  L  V  *  C  T  I  S  P  R
                                             STOP

SqrP2.abi         -------------------------------------------------------
P1.abi            -------------------------------------------------------
P3.abi            -------------------------------------------------------
P4.abi            -------------------------------------------------------
P6.abi            -------------------------------------------------------
P8.abi            -------------------------------------------------------
P10.abi           -------------------------------------------------------
P12.abi           TATAAGGACGATGACGATAAGTAACATTTGGTTTAGTGTACAATATCTCCTCGAG
```

```
                                                                                               SacI                    KpnI
                     BglII                                   NotI           HindIII      EcoICRI EcoRI           Acc65I
ref/ΔDUF(630-709) AGATCAGATCTTTGTCGATCCTACCATCCACTCGACACACCCGCCAGCGGCCGCTGCCAAGCTTCCGAGCTCTCGAATTCAAAGGAGGTACCCACCCTTG

SqrP2.abi         ----------------------------------------------------------------------------------------------------
P1.abi            ----------------------------------------------------------------------------------------------------
P3.abi            ----------------------------------------------------------------------------------------------------
P4.abi            ----------------------------------------------------------------------------------------------------
P6.abi            ----------------------------------------------------------------------------------------------------
P8.abi            ----------------------------------------------------------------------------------------------------
P10.abi           ----------------------------------------------------------------------------------------------------
P12.abi           ----------------------------------------------------------------------------------------------------
```

```
                     DraI                                            Bpu10I          StuI     PstI
ref/ΔDUF(630-709) TTTAAACTTTAAGAGGAGGGCCACCATGAAAAGCCCTGCTTTGCAACCCCTCAGCATGGCAGGCCTGCAGCTCATGACCCCTGCTTCCTCACCAATGGGT
                                          M  K  S  P  A  L  Q  P  L  S  M  A  G  L  Q  L  M  T  P  A  S  S  P  M  G
                                           START

SqrP2.abi         --------TTAAGAGGAGGGCCACCATGAAAAGCCCTGCTTTGCAACCCCTCAGCATGGCAGGCCTGCAGCTCATGACCCCTGCTTCCTCACCAATGGGT
P1.abi            -------------------------------------------------------------------CAGCTCATGACCCCTGCTTCCTCACCAATGGGT
P3.abi            ----------------------------------------------------------------------------------------------------
P4.abi            ----------------------------------------------------------------------------------------------------
P6.abi            ----------------------------------------------------------------------------------------------------
P8.abi            ----------------------------------------------------------------------------------------------------
P10.abi           ----------------------------------------------------------------------------------------------------
P12.abi           ----------------------------------------------------------------------------------------------------
```

```
                   NcoI
ref/ΔDUF(630-709)  CCTTTCTTTGGACTGCCATGGCAACAAGAAGCAATTCATGATAACATTTATACGCCAAGAAAATATCAGGTTGAACTGCTTGAAGCAGCTCTGGATCATA
                    P  F  F  G  L  P  W  Q  Q  E  A  I  H  D  N  I  Y  T  P  R  K  Y  Q  V  E  L  L  E  A  A  L  D  H

SqrP2.abi          CCTTTCTTTGGACTGCCATGGCAACAAGAAGCAATTCATGATAACATTTATACGCCAAGAAAATATCAGGTTGAACTGCTTGAAGCAGCTCTGGATCATA
P1.abi             CCTTTCTTTGGACTGCCATGGCAACAAGAAGCAATTCATGATAACATTTATACGCCAAGAAAATATCAGGTTGAACTGCTTGAAGCAGCTCTGGATCATA
P3.abi             ----------------------------------------------------------------------------------------------------
P4.abi             ----------------------------------------------------------------------------------------------------
P6.abi             ----------------------------------------------------------------------------------------------------
P8.abi             ----------------------------------------------------------------------------------------------------
P10.abi            ----------------------------------------------------------------------------------------------------
P12.abi            ----------------------------------------------------------------------------------------------------
```

```
                   DraI      Bpu10I                 ScaI
ref/ΔDUF(630-709)  ATACCATCGTCTGTTTAAACACTGGCTCAGGGAAGACATTTATTGCAGTACTACTCACTAAAGAGCTGTCCTATCAGATCAGGGGAGACTTCAGCAGAAA
                   N  T  I  V  C  L  N  T  G  S  G  K  T  F  I  A  V  L  L  T  K  E  L  S  Y  Q  I  R  G  D  F  S  R  N

SqrP2.abi          ATACCATCGTCTGTTTAAACACTGGCTCAGGGAAGACATTTATTGCAGTACTACTCACTAAAGAGCTGTCCTATCAGATCAGGGGAGACTTCAGCAGAAA
P1.abi             ATACCATCGTCTGTTTAAACACTGGCTCAGGGAAGACATTTATTGCAGTACTACTCACTAAAGAGCTGTCCTATCAGATCAGGGGAGACTTCAGCAGAAA
P3.abi             ----------------------------------------------------------------------------------------------------
P4.abi             ----------------------------------------------------------------------------------------------------
P6.abi             ----------------------------------------------------------------------------------------------------
P8.abi             ----------------------------------------------------------------------------------------------------
P10.abi            ----------------------------------------------------------------------------------------------------
P12.abi            ----------------------------------------------------------------------------------------------------
```

```
                                                                           |PvuII             |BglII
ref/ΔDUF(630-709) TGGAAAAAGGACGGTGTTCTTGGTCAACTCTGCAAACCAGGTTGCTCAACAAGTGTCAGCTGTCAGAACTCATTCAGATCTCAAGGTTGGGGAATACTCA
                    G  K  R  T  V  F  L  V  N  S  A  N  Q  V  A  Q  Q  V  S  A  V  R  T  H  S  D  L  K  V  G  E  Y  S

SqrP2.abi         TGGAAAAAGGACGGTGTTCTTGGTCAACTCTGCAAACCAGGTTGCTCAACAAGTGTCAGCTGTCAGAACTCATTCAGATCTCAAGGTTGGG---------
P1.abi            TGGAAAAAGGACGGTGTTCTTGGTCAACTCTGCAAACCAGGTTGCTCAACAAGTGTCAGCTGTCAGAACTCATTCAGATCTCAAGGTTGGGGAATACTCA
P3.abi            ----------------------------------------------------------------------------------------------------
P4.abi            ----------------------------------------------------------------------------------------------------
P6.abi            ----------------------------------------------------------------------------------------------------
P8.abi            ----------------------------------------------------------------------------------------------------
P10.abi           ----------------------------------------------------------------------------------------------------
P12.abi           ----------------------------------------------------------------------------------------------------
```

```
                                    |NsiI
ref/ΔDUF(630-709) AACCTAGAAGTAAATGCATCTTGGACAAAAGAGAGATGGAACCAAGAGTTTACTAAGCACCAGGTTCTCATTATGACTTGCTATGTCGCCTTGAATGTTT
                   N  L  E  V  N  A  S  W  T  K  E  R  W  N  Q  E  F  T  K  H  Q  V  L  I  M  T  C  Y  V  A  L  N  V

SqrP2.abi         ----------------------------------------------------------------------------------------------------
P1.abi            AACCTAGAAGTAAATGCATCTTGGACAAAAGAGAGATGGAACCAAGAGTTTACTAAGCACCAGGTTCTCATTATGACTTGCTATGTCGCCTTGAATGTTT
P3.abi            ----------------------------------------------------------------------------------------------------
P4.abi            ----------------------------------------------------------------------------------------------------
P6.abi            ----------------------------------------------------------------------------------------------------
P8.abi            ----------------------------------------------------------------------------------------------------
P10.abi           ----------------------------------------------------------------------------------------------------
P12.abi           ----------------------------------------------------------------------------------------------------
```

```
ref/ΔDUF(630-709) TGAAAAATGGTTACTTATCACTGTCAGACATTAACCTTTTGGTGTTTGATGAGTGTCATCTTGCAATCCTAGACCACCCCTATCGAGAAATTATGAAGCT
                  L K N G Y L S L S D I N L L V F D E C H L A I L D H P Y R E I M K L

SqrP2.abi         -----------------------------------------------------------------------------------------------------
P1.abi            TGAAAAATGGTTACTTATCACTGTCAGACATTAACCTTTTGGTGTTTGATGAGTGTCATCTTGCAATCCTAGACCACCCCTATCGAGAAATTATGAAGCT
P3.abi            -----------------------------------------------------------------------------------------------------
P4.abi            -----------------------------------------------------------------------------------------------------
P6.abi            -----------------------------------------------------------------------------------------------------
P8.abi            -----------------------------------------------------------------------------------------------------
P10.abi           -----------------------------------------------------------------------------------------------------
P12.abi           -----------------------------------------------------------------------------------------------------
```

```
                                                                       DraI
ref/ΔDUF(630-709) CTGTGAAAATTGTCCATCATGTCCTCGCATTTTGGGACTAACTGCTTCCATTTTAAATGGGAAATGTGATCCAGAGGAATTGGAAGAAAAGATTCAGAAA
                  C E N C P S C P R I L G L T A S I L N G K C D P E E L E E K I Q K

SqrP2.abi         -----------------------------------------------------------------------------------------------------
P1.abi            CTGTGAAAATTGTCCATCATGTCCTCGCATTTTGGGACTAACTGCTTCCATTTTAAATGGGAAATGTGATCCAGAGGAATTGGAAGAAAAGATTCAGAAA
P3.abi            -----------------------------------------------------------------------------------------------------
P4.abi            -----------------------------------------------------------------------------------------------------
P6.abi            -----------------------------------------------------------------------------------------------------
P8.abi            -----------------------------------------------------------------------------------------------------
P10.abi           -----------------------------------------------------------------------------------------------------
P12.abi           -----------------------------------------------------------------------------------------------------
```

```
                    | AflII
ref/ΔDUF(630-709) CTAGAGAAAATTCTTAAGAGTAATGCTGAAACTGCAACTGACCTGGTGGTCTTAGACAGGTATACTTCTCAGCCATGTGAGATTGTGGTGGATTGTGGAC
                   L  E  K  I  L  K  S  N  A  E  T  A  T  D  L  V  V  L  D  R  Y  T  S  Q  P  C  E  I  V  V  D  C  G

SqrP2.abi         ----------------------------------------------------------------------------------------------------
P1.abi            CTAGAGAAAATTCTTAAGAGTAATGCTGAAACTGCAACTGACCTGGTGGTCTTAGACAGGTATACTTCTCAGCCATGTGAGATTGTGGTGGATTGTGGAC
P3.abi            ----------------------------------------------------------------------------------------------------
P4.abi            ----------------------------------------------------------------------------------------------------
P6.abi            ----------------------------------------------------------------------------------------------------
P8.abi            ----------------------------------------------------------------------------------------------------
P10.abi           ----------------------------------------------------------------------------------------------------
P12.abi           ----------------------------------------------------------------------------------------------------
```

```
ref/ΔDUF(630-709) CATTTACTGACAGAAGTGGGCTTTATGAAAGACTGCTGATGGAATTAGAAGAAGCACTTAATTTTATCAATGATTGTAATATATCTGTACATTCAAAAGA
                  P  F  T  D  R  S  G  L  Y  E  R  L  L  M  E  L  E  E  A  L  N  F  I  N  D  C  N  I  S  V  H  S  K  E

SqrP2.abi         ----------------------------------------------------------------------------------------------------
P1.abi            CATTTACTGACAGAAGTGGGCTTTATGAAAGACTGCTGATGGAATTAGAAGAAGCACTTAATTTTATCAATGATTGTAATATATCTGTACATTCAAAAGA
P3.abi            ----------------------------------------------------------------------------------------------------
P4.abi            ----------------------------------------------------------------------------------------------------
P6.abi            ----------------------------------------------------------------------------------------------------
P8.abi            ----------------------------------------------------------------------------------------------------
P10.abi           ----------------------------------------------------------------------------------------------------
P12.abi           ----------------------------------------------------------------------------------------------------
```

```
ref/ΔDUF(630-709) AAGAGATTCTACTTTAATTTCGAAACAGATACTATCAGACTGTCGTGCCGTATTGGTAGTTCTGGGACCCTGGTGTGCAGATAAAGTAGCTGGAATGATG
                   R  D  S  T  L  I  S  K  Q  I  L  S  D  C  R  A  V  L  V  V  L  G  P  W  C  A  D  K  V  A  G  M  M

SqrP2.abi         ---------------------------------------------------------------------------------------------------
P1.abi            AAGAGATTCTACTTTAATTTCGAAACAGATACTATCAGACTGTCGTGCCGTATTGGTAGTTCTGGGACCCTGGTGTGCAGATAAAGTAGCTGGAATGATG
P3.abi            ---------------------------------------------------------------------------------------------------
P4.abi            ---------------------------------------------------------------------------------------------------
P6.abi            ---------------------------------------------------------------------------------------------------
P8.abi            ---------------------------------------------------------------------------------------------------
P10.abi           ---------------------------------------------------------------------------------------------------
P12.abi           ---------------------------------------------------------------------------------------------------
```

```
ref/ΔDUF(630-709) GTAAGAGAACTACAGAAATACATCAAACATGAGCAAGAGGAGCTGCACAGGAAATTTTTATTGTTTACAGACACTTTCCTAAGGAAAATACATGCACTAT
                  V  R  E  L  Q  K  Y  I  K  H  E  Q  E  E  L  H  R  K  F  L  L  F  T  D  T  F  L  R  K  I  H  A  L

SqrP2.abi         ---------------------------------------------------------------------------------------------------
P1.abi            GTAAGAGAACTACAGAAATACATCAAACATGAGCAAGAGGAGCTGCACAGGAAATTTTTATTGTTTACAGAC---------------------------
P3.abi            -----------------ATACATCAAACATGAGCAAGAGGAGCTGCACAGGAAATTTTTATTGTTTACAGACACTTTCCTAAGGAAAATACATGCACTAT
P4.abi            ---------------------------------------------------------------------------------------------------
P6.abi            ---------------------------------------------------------------------------------------------------
P8.abi            ---------------------------------------------------------------------------------------------------
P10.abi           ---------------------------------------------------------------------------------------------------
P12.abi           ---------------------------------------------------------------------------------------------------
```

```
                  | DraIII                                                                                  | NdeI
ref/ΔDUF(630-709) GTGAAGAGCACTTCTCACCTGCCTCACTTGACCTGAAATTTGTAACTCCTAAAGTAATCAAACTGCTCGAAATCTTACGCAAATATAAACCATATGAGCG
                  C  E  E  H  F  S  P  A  S  L  D  L  K  F  V  T  P  K  V  I  K  L  L  E  I  L  R  K  Y  K  P  Y  E  R

SqrP2.abi         -------- (no sequence)
P1.abi            -------- (no sequence)
P3.abi            GTGAAGAGCACTTCTCACCTGCCTCACTTGACCTGAAATTTGTAACTCCTAAAGTAATCAAACTGCTCGAAATCTTACGCAAATATAAACCATATGAGCG
P4.abi            -------- (no sequence)
P6.abi            -------- (no sequence)
P8.abi            -------- (no sequence)
P10.abi           -------- (no sequence)
P12.abi           -------- (no sequence)
```

```
ref/ΔDUF(630-709) ACAGCAGTTTGAAAGCGTTGAGTGGTATAATAATAGAAATCAGGATAATTATGTGTCATGGAGTGATTCTGAGGATGATGATGAGGATGAAGAAATTGAA
                   Q  Q  F  E  S  V  E  W  Y  N  N  R  N  Q  D  N  Y  V  S  W  S  D  S  E  D  D  D  E  D  E  E  I  E

SqrP2.abi         -------- (no sequence)
P1.abi            -------- (no sequence)
P3.abi            ACAGCAGTTTGAAAGCGTTGAGTGGTATAATAATAGAAATCAGGATAATTATGTGTCATGGAGTGATTCTGAGGATGATGATGAGGATGAAGAAATTGAA
P4.abi            -------- (no sequence)
P6.abi            -------- (no sequence)
P8.abi            -------- (no sequence)
P10.abi           -------- (no sequence)
P12.abi           -------- (no sequence)
```

```
ref/ΔDUF(630-709) GAAAAAGAGAAGCCAGAGACAAATTTTCCTTCTCCTTTTACCAACATTTTGTGCGGAATTATTTTTGTGGAAAGAAGATACACAGCAGTTGTCTTAAACA
                   E  K  E  K  P  E  T  N  F  P  S  P  F  T  N  I  L  C  G  I  I  F  V  E  R  R  Y  T  A  V  V  L  N

SqrP2.abi         ----------------------------------------------------------------------------------------------------
P1.abi            ----------------------------------------------------------------------------------------------------
P3.abi            GAAAAAGAGAAGCCAGAGACAAATTTTCCTTCTCCTTTTACCAACATTTTGTGCGGAATTATTTTTGTGGAAAGAAGATACACAGCAGTTGTCTTAAACA
P4.abi            ----------------------------------------------------------------------------------------------------
P6.abi            ----------------------------------------------------------------------------------------------------
P8.abi            ----------------------------------------------------------------------------------------------------
P10.abi           ----------------------------------------------------------------------------------------------------
P12.abi           ----------------------------------------------------------------------------------------------------
```

```
ref/ΔDUF(630-709) GATTGATAAAGGAAGCTGGCAAACAAGATCCAGAGCTGGCTTATATCAGTAGCAATTTCATAACTGGACATGGCATTGGGAAGAATCAGCCTCGCAACAA
                  R  L  I  K  E  A  G  K  Q  D  P  E  L  A  Y  I  S  S  N  F  I  T  G  H  G  I  G  K  N  Q  P  R  N  K

SqrP2.abi         ----------------------------------------------------------------------------------------------------
P1.abi            ----------------------------------------------------------------------------------------------------
P3.abi            GATTGATAAAGGAAGCTGGCAAACAAGATCCAGAGCTGGCTTATATCAGTAGCAATTTCATAACTGGACATGGCATTGGGAAGAATCAGCCTCGCAACAA
P4.abi            ------------------------AAGATCCAGAGCTGGCTTATATCAGTAGCAATTTCATAACTGGACATGGCATTGGGAAGAATCAGCCTCGCAACAA
P6.abi            ----------------------------------------------------------------------------------------------------
P8.abi            ----------------------------------------------------------------------------------------------------
P10.abi           ----------------------------------------------------------------------------------------------------
P12.abi           ----------------------------------------------------------------------------------------------------
```

```
                   EcoRI
ref/ΔDUF(630-709) ACAGATGGAAGCAGAATTCAGAAAACAGGAAGAGGTACTTAGGAAATTTCGAGCACATGAGACCAACCTGCTTATTGCAACAAGTATTGTAGAAGAGGGT
                   Q  M  E  A  E  F  R  K  Q  E  E  V  L  R  K  F  R  A  H  E  T  N  L  L  I  A  T  S  I  V  E  E  G

SqrP2.abi         ---------------------------------------------------------------------------------------------------
P1.abi            ---------------------------------------------------------------------------------------------------
P3.abi            ACAGATGGAAGCAGAATTCAGAAAACAGGAAGAGGTACTTAGGAAATTTCGAGCACATGAGACCAACCTGCTTATTGCAACAAGTATTGTAGAAGAGGGT
P4.abi            ACAGATGGAAGCAGAATTCAGAAAACAGGAAGAGGTACTTAGGAAATTTCGAGCACATGAGACCAACCTGCTTATTGCAACAAGTATTGTAGAAGAGGGT
P6.abi            ---------------------------------------------------------------------------------------------------
P8.abi            ---------------------------------------------------------------------------------------------------
P10.abi           ---------------------------------------------------------------------------------------------------
P12.abi           ---------------------------------------------------------------------------------------------------
```

```
                                                                ClaI*
ref/ΔDUF(630-709) GTTGATATACCAAAATGCAACTTGGTGGTTCGTTTTGATTTGCCCACAGAATATCGATCCTATGTTCAATCTAAAGGAAGAGCAAGGGCACCCATCTCTA
                   V  D  I  P  K  C  N  L  V  V  R  F  D  L  P  T  E  Y  R  S  Y  V  Q  S  K  G  R  A  R  A  P  I  S

SqrP2.abi         ---------------------------------------------------------------------------------------------------
P1.abi            ---------------------------------------------------------------------------------------------------
P3.abi            GTTGATATACCAAAATGCAACTTGGTGGTTCGTTTTGATTTGCCCACAGAATATCGATCCTATGTTCAATCTAAAGGAAGAGCAAGGGCACCCATCTCTA
P4.abi            GTTGATATACCAAAATGCAACTTGGTGGTTCGTTTTGATTTGCCCACAGAATATCGATCCTATGTTCAATCTAAAGGAAGAGCAAGGGCACCCATCTCTA
P6.abi            ---------------------------------------------------------------------------------------------------
P8.abi            ---------------------------------------------------------------------------------------------------
P10.abi           ---------------------------------------------------------------------------------------------------
P12.abi           ---------------------------------------------------------------------------------------------------
```

	BglII	
ref/ΔDUF(630-709)	ATTATATAATGTTAGCGGATACAGACAAAATAAAAAGTTTTGAAGAAGACCTTAAAACCTACAAAGCTATTGAAAAGATCTTGAGAAACAAGTGTTCCAA	
	N Y I M L A D T D K I K S F E E D L K T Y K A I E K I L R N K C S K	
SqrP2.abi		
P1.abi		
P3.abi	ATTATATAATGTTAGCGGATACAGACAAAATAAAAAGTTTTGAAGAAGACCTTAAAACCTACAA	
P4.abi	ATTATATAATGTTAGCGGATACAGACAAAATAAAAAGTTTTGAAGAAGACCTTAAAACCTACAAAGCTATTGAAAAGATCTTGAGAAACAAGTGTTCCAA	
P6.abi		
P8.abi		
P10.abi		
P12.abi		

	NdeI StuI	
ref/ΔDUF(630-709)	GTCGGTTGATACTGGTGAGACTGACATTGATCCTGTCATGGATGATGATGACGTTTTCCCACCATATGTGTTGAGGCCTGACGATGGTGGTCCACGAGTC	
	S V D T G E T D I D P V M D D D D V F P P Y V L R P D D G G P R V	
SqrP2.abi		
P1.abi		
P3.abi		
P4.abi	GTCGGTTGATACTGGTGAGACTGACATTGATCCTGTCATGGATGATGATGACGTTTTCCCACCATATGTGTTGAGGCCTGACGATGGTGGTCCACGAGTC	
P6.abi		
P8.abi		
P10.abi		
P12.abi		

```
ref/ΔDUF(630-709) ACAATCAACACGGCCGACCATTTGATGCCAGTTGGGAAAGAGACTGTTAAATATGAAGAGGAGCTTGATTTGCATGATGAAGAAGAGACCAGTGTTCCAG
                   T  I  N  T  A  D  H  L  M  P  V  G  K  E  T  V  K  Y  E  E  E  L  D  L  H  D  E  E  E  T  S  V  P

SqrP2.abi         ----------------------------------------------------------------------------------------------------
P1.abi            ----------------------------------------------------------------------------------------------------
P3.abi            ----------------------------------------------------------------------------------------------------
P4.abi            ACAATCAACACGGCCGACCATTTGATGCCAGTTGGGAAAGAGACTGTTAAATATGAAGAGGAGCTTGATTTGCATGATGAAGAAGAGACCAGTGTTCCAG
P6.abi            ----------------------------------------------------------------------------------------------------
P8.abi            ----------------------------------------------------------------------------------------------------
P10.abi           ----------------------------------------------------------------------------------------------------
P12.abi           ----------------------------------------------------------------------------------------------------
```

```
                                                                                          |BclI*
ref/ΔDUF(630-709) GAAGACCAGGTTCCACGAAACGAAGGCAGTGCTACCCAAAAGCAATTCCAGAGTGTTTGAGGGATAGTTATCCCAGACCTGATCAGCCCTGTTACCTGTA
                  G  R  P  G  S  T  K  R  R  Q  C  Y  P  K  A  I  P  E  C  L  R  D  S  Y  P  R  P  D  Q  P  C  Y  L  Y

SqrP2.abi         ----------------------------------------------------------------------------------------------------
P1.abi            ----------------------------------------------------------------------------------------------------
P3.abi            ----------------------------------------------------------------------------------------------------
P4.abi            GAAGACCAGGTTCCACGAAACGAAGGCAGTGCTACCCAAAAGCAATTCCAGAGTGTTTGAGGGATAGTTATCCCAGACCTGATCAGCCCTGTTACCTGTA
P6.abi            ----------------------------------------------------------------------------------------------------
P8.abi            ----------------------------------------------------------------------------------------------------
P10.abi           ----------------------------------------------------------------------------------------------------
P12.abi           ----------------------------------------------------------------------------------------------------
```

```
ref/ΔDUF(630-709) TGTGATAGGAATGGTTTTAACTACACCTTTACCTGATGAACTCAACTTTAGAAGGCGGAAGCTCTATCCTCCTGAAGATACCACAAGATGCTTTGGAATA
                    V  I  G  M  V  L  T  T  P  L  P  D  E  L  N  F  R  R  R  K  L  Y  P  P  E  D  T  T  R  C  F  G  I

SqrP2.abi         ----------------------------------------------------------------------------------------------------
P1.abi            ----------------------------------------------------------------------------------------------------
P3.abi            ----------------------------------------------------------------------------------------------------
P4.abi            TGTGATAGGAATGGTTTTAACTACACCTTTACCTGATGAACTCAACTTTAGAAGGCGGAAGCTCTATCCTCCTGAAGATACCACAAGATGCTTTGGAATA
P6.abi            ---------------------------------------------------------------------------------CACAAGATGCTTTGGAATA
P8.abi            ----------------------------------------------------------------------------------------------------
P10.abi           ----------------------------------------------------------------------------------------------------
P12.abi           ----------------------------------------------------------------------------------------------------
```

```
                                                                              |BstEII
ref/ΔDUF(630-709) CTGACGGCCAAACCCATACCTCAGATTCCACACTTTCCTGTGTACACACGCTCTGGAGAGGTTACCATATCCATTGAGTTGAAGAAGTCTGGTTTCATGT
                   L  T  A  K  P  I  P  Q  I  P  H  F  P  V  Y  T  R  S  G  E  V  T  I  S  I  E  L  K  K  S  G  F  M

SqrP2.abi         ----------------------------------------------------------------------------------------------------
P1.abi            ----------------------------------------------------------------------------------------------------
P3.abi            ----------------------------------------------------------------------------------------------------
P4.abi            CTGACGGCCAAACCCATACCTCAGATTCCACACTTTCCTGTGTACACACGCTCTGGAGAGGTTACCATATCCATTGAGTTGAAGAAGTCTGGTTTCATGT
P6.abi            CTGACGGCCAAACCCATACCTCAGATTCCACACTTTCCTGTGTACACACGCTCTGGAGAGGTTACCATATCCATTGAGTTGAAGAAGTCTGGTTTCATGT
P8.abi            ----------------------------------------------------------------------------------------------------
P10.abi           ----------------------------------------------------------------------------------------------------
P12.abi           ----------------------------------------------------------------------------------------------------
```

```
                                                                                                   DraI
ref/ΔDUF(630-709) TGTCTCTACAAATGCTTGAGTTGATTACAAGACTTCACCAGTATATATTCTCACATATTCTTCGGCTTGAAAAACCTGCACTAGAATTTAAACCTACAGA
                  L  S  L  Q  M  L  E  L  I  T  R  L  H  Q  Y  I  F  S  H  I  L  R  L  E  K  P  A  L  E  F  K  P  T  D

SqrP2.abi         ----------------------------------------------------------------------------------------------------
P1.abi            ----------------------------------------------------------------------------------------------------
P3.abi            ----------------------------------------------------------------------------------------------------
P4.abi            TGTCTCTACAAATGCTTGAGTTGATTACAAGACTTCACCAGTATATATTCTCACATATTCTTCGGCTTGAAAAACCTGCACTAGAATTTAAACCTACAGA
P6.abi            TGTCTCTACAAATGCTTGAGTTGATTACAAGACTTCACCAGTATATATTCTCACATATTCTTCGGCTTGAAAAACCTGCACTAGAATTTAAACCTACAGA
P8.abi            ----------------------------------------------------------------------------------------------------
P10.abi           ----------------------------------------------------------------------------------------------------
P12.abi           ----------------------------------------------------------------------------------------------------
```

```
                                                                 BstXI             DraI
ref/ΔDUF(630-709) CGCTGATTCAGCATACTGTGTTCTACCTCTTAATGTTGTTAATGACTCCAGCACTTTGGATATTGACTTTAAATTCATGGAAGATATTGAGAAGTCTGAA
                    A  D  S  A  Y  C  V  L  P  L  N  V  V  N  D  S  S  T  L  D  I  D  F  K  F  M  E  D  I  E  K  S  E

SqrP2.abi         ----------------------------------------------------------------------------------------------------
P1.abi            ----------------------------------------------------------------------------------------------------
P3.abi            ----------------------------------------------------------------------------------------------------
P4.abi            CGCTGATTCAGCATACTGTGTTCTACCTCTTAATGTTGTTAATGACTCCAGCACTTTGGATATTGACTTTAAATTCATGGAAGATATTGAGAAGTCTGAA
P6.abi            CGCTGATTCAGCATACTGTGTTCTACCTCTTAATGTTGTTAATGACTCCAGCACTTTGGATATTGACTTTAAATTCATGGAAGATATTGAGAAGTCTGAA
P8.abi            ----------------------------------------------------------------------------------------------------
P10.abi           ----------------------------------------------------------------------------------------------------
P12.abi           ----------------------------------------------------------------------------------------------------
```

```
                         BsmI                                        DraI                                  EcoRV
ref/ΔDUF(630-709) GCTCGCATAGGCATTCCCAGTACAAAGTATACAAAAGAAACACCCTTTGTTTTTAAATTAGAAGATTACCAAGATGCCGTTATCATTCCAAGATATCGCA
                   A  R  I  G  I  P  S  T  K  Y  T  K  E  T  P  F  V  F  K  L  E  D  Y  Q  D  A  V  I  I  P  R  Y  R

SqrP2.abi         ----------------------------------------------------------------------------------------------------
P1.abi            ----------------------------------------------------------------------------------------------------
P3.abi            ----------------------------------------------------------------------------------------------------
P4.abi            GCTCGCATAGGCATTCCCAGTACAAAGT------------------------------------------------------------------------
P6.abi            GCTCGCATAGGCATTCCCAGTACAAAGTATACAAAAGAAACACCCTTTGTTTTTAAATTAGAAGATTACCAAGATGCCGTTATCATTCCAAGATATCGCA
P8.abi            ----------------------------------------------------------------------------------------------------
P10.abi           ----------------------------------------------------------------------------------------------------
P12.abi           ----------------------------------------------------------------------------------------------------
```

```
                       BclI*    ClaI                                                                                 S
ref/ΔDUF(630-709) ATTTTGATCAGCCTCATCGATTTTATGTAGCTGATGTGTACACTGATCTTACCCCACTCAGTAAATTTCCTTCCCCTGAGTATGAAACTTTTGCAGAATA
                  N  F  D  Q  P  H  R  F  Y  V  A  D  V  Y  T  D  L  T  P  L  S  K  F  P  S  P  E  Y  E  T  F  A  E  Y

SqrP2.abi         ----------------------------------------------------------------------------------------------------
P1.abi            ----------------------------------------------------------------------------------------------------
P3.abi            ----------------------------------------------------------------------------------------------------
P4.abi            ----------------------------------------------------------------------------------------------------
P6.abi            ATTTTGATCAGCCTCATCGATTTTATGTAGCTGATGTGTACACTGATCTTACCCCACTCAGTAAATTTCCTTCCCCTGAGTATGAAACTTTTGCAGAATA
P8.abi            ----------------------------------------------------------------------------------------------------
P10.abi           ----------------------------------------------------------------------------------------------------
P12.abi           ----------------------------------------------------------------------------------------------------
```

```
                  SspI
ref/ΔDUF(630-709) TTATAAAACAAAGTACAACCTTGACCTAACCAATCTCAACCAGCCACTGCTGGATGTGGACCACACATCTTCAAGACTTAATCTTTTGACACCTCGACAT
                   Y  K  T  K  Y  N  L  D  L  T  N  L  N  Q  P  L  L  D  V  D  H  T  S  S  R  L  N  L  L  T  P  R  H

SqrP2.abi         ----------------------------------------------------------------------------------------------------
P1.abi            ----------------------------------------------------------------------------------------------------
P3.abi            ----------------------------------------------------------------------------------------------------
P4.abi            ----------------------------------------------------------------------------------------------------
P6.abi            TTATAAAACAAAGTACAACCTTGACCTAACCAATCTCAACCAGCCACTGCTGGATGTGGACCACACATCTTCAAGACTTAATCTTTTGACACCTCGACAT
P8.abi            ----------------------------------------------------------------------------------------------------
P10.abi           ----------------------------------------------------------------------------------------------------
P12.abi           ----------------------------------------------------------------------------------------------------
```

```
                                    Eco47III                                                      PstI
ref/ΔDUF(630-709) TTGAATCAGAAGGGGAAAGCGCTTCCTTTAAGCAGTGCTGAGAAGAGGAAAGCCAAATGGGAAAGTCTGCAGAATAAACAGATACTGGTTCCAGAACTCT
                  L  N  Q  K  G  K  A  L  P  L  S  S  A  E  K  R  K  A  K  W  E  S  L  Q  N  K  Q  I  L  V  P  E  L

SqrP2.abi         ----------------------------------------------------------------------------------------------------
P1.abi            ----------------------------------------------------------------------------------------------------
P3.abi            ----------------------------------------------------------------------------------------------------
P4.abi            ----------------------------------------------------------------------------------------------------
P6.abi            TTGAATCAGAAGGGGAAAGCGCTTCCTTTAAGCAGTGCTGAGAAGAGGAAAGCCAAATGGGAAAGTCTGCAGAATAAACAGATACTGGTTCCAGAACTCT
P8.abi            ----------------------------------------------------------------------------------------------------
P10.abi           ----------------------------------------------------------------------------------------------------
P12.abi           ----------------------------------------------------------------------------------------------------
```

```
                                                                                                      PstI
ref/ΔDUF(630-709) GTGCTATACATCCAATTCCAGCATCACTGTGGAGAAAAGCTGTTTGTCTCCCCAGCATACTTTATCGCCTTCACTGCCTTTTGACTGCAGAGGAGCTAAG
                  C  A  I  H  P  I  P  A  S  L  W  R  K  A  V  C  L  P  S  I  L  Y  R  L  H  C  L  L  T  A  E  E  L  R

SqrP2.abi         ----------------------------------------------------------------------------------------------------
P1.abi            ----------------------------------------------------------------------------------------------------
P3.abi            ----------------------------------------------------------------------------------------------------
P4.abi            ----------------------------------------------------------------------------------------------------
P6.abi            GTGCTATACATCCAATTCCAGCATCACTGTGGAGAAAAGCTGTTTGTCTCCCCAGCATACTTTATCGCCTTCACTGCCTTTTGACTGCAGAGGAGCTAAG
P8.abi            ----------------------------------------------------------------------------------------------------
P10.abi           ----------------------------------------------------------------------------------------------------
P12.abi           ----------------------------------------------------------------------------------------------------
```

```
ref/ΔDUF(630-709) AGCCCAGACTGCCAGCGATGCTGGCGTGGGAGTCAGATCACTTCCTGCGGATTTTAGATACCCTAACTTAGACTTCGGGTGGAAAAAATCTATTGACAGC
                    A  Q  T  A  S  D  A  G  V  G  V  R  S  L  P  A  D  F  R  Y  P  N  L  D  F  G  W  K  K  S  I  D  S

SqrP2.abi         ----------------------------------------------------------------------------------------------------
P1.abi            ----------------------------------------------------------------------------------------------------
P3.abi            ----------------------------------------------------------------------------------------------------
P4.abi            ----------------------------------------------------------------------------------------------------
P6.abi            AGCCCAGACTGCCAGCGATGCTGGCGTGGGAGTCAGATCACTTCCTGCGGATTTTAGATACCCTAACTTAGACTTCGGGTGGAAAAAATCTATTGACAGC
P8.abi            ---------------------------------------------------------------------------------------AATCTATTGACAGC
P10.abi           ----------------------------------------------------------------------------------------------------
P12.abi           ----------------------------------------------------------------------------------------------------
```

```
                                                PvuII                                       MunI
ref/ΔDUF(630-709) AAATCTTTCATCTCAATTTCTAACTCCTCTTCAGCTGAAAATGATAATTACTGTAAGCACAGCACAATTGTCCCTGAAAATGCTGCACATCAAGGTGCTA
                   K  S  F  I  S  I  S  N  S  S  S  A  E  N  D  N  Y  C  K  H  S  T  I  V  P  E  N  A  A  H  Q  G  A

SqrP2.abi         ----------------------------------------------------------------------------------------------------
P1.abi            ----------------------------------------------------------------------------------------------------
P3.abi            ----------------------------------------------------------------------------------------------------
P4.abi            ----------------------------------------------------------------------------------------------------
P6.abi            AAATCTTTCATCTCAATTTCTAACTCCTCTTCAGCTGAAAATGATAATTACTGTAAGCACAGCACAATTGTCCCTGAAAATGCTGCACATCAAGGTGCTA
P8.abi            AAATCTTTCATCTCAATTTCTAACTCCTCTTCAGCTGAAAATGATAATTACTGTAAGCACAGCACAATTGTCCCTGAAAATGCTGCACATCAAGGTGCTA
P10.abi           ----------------------------------------------------------------------------------------------------
P12.abi           ----------------------------------------------------------------------------------------------------
```

```
                                                                       AclI
                              XbaI                                  PstI                                               Bg
ref/ΔDUF(630-709) ATAGAACCTCCTCTCTAGAAAATCATGACCAAATGTCTGTGAACTGCAGAACGTTGCTCAGCGAGTCCCCTGGTAAGCTCCACGTTGAAGTTTCAGCAGA
                  N  R  T  S  S  L  E  N  H  D  Q  M  S  V  N  C  R  T  L  L  S  E  S  P  G  K  L  H  V  E  V  S  A  D

SqrP2.abi         ----------------------------------------------------------------------------------------------------
P1.abi            ----------------------------------------------------------------------------------------------------
P3.abi            ----------------------------------------------------------------------------------------------------
P4.abi            ----------------------------------------------------------------------------------------------------
P6.abi            ATAGAACCTCCTCTCTAGAAAATCATGACCAAATGTCTGTGAACTGCAGAACGTTGCTCAGCGAGTCCCCTGGTAAGCTCCACGTTGAAGTTTCAGCAGA
P8.abi            ATAGAACCTCCTCTCTAGAAAATCATGACCAAATGTCTGTGAACTGCAGAACGTTGCTCAGCGAGTCCCCTGGTAAGCTCCACGTTGAAGTTTCAGCAGA
P10.abi           ----------------------------------------------------------------------------------------------------
P12.abi           ----------------------------------------------------------------------------------------------------
```

```
                    ;lII           VspI
ref/ΔDUF(630-709)  TCTTACAGCAATTAATGGTCTTTCTTACAATCAAAATCTCGCCAATGGCAGTTATGATTTAGCTAACAGAGACTTTTGCCAAGGAAATCAGCTAAATTAC
                    L  T  A  I  N  G  L  S  Y  N  Q  N  L  A  N  G  S  Y  D  L  A  N  R  D  F  C  Q  G  N  Q  L  N  Y

SqrP2.abi          ----------------------------------------------------------------------------------------------------
P1.abi             ----------------------------------------------------------------------------------------------------
P3.abi             ----------------------------------------------------------------------------------------------------
P4.abi             ----------------------------------------------------------------------------------------------------
P6.abi             TCTTA-----------------------------------------------------------------------------------------------
P8.abi             TCTTACAGCAATTAATGGTCTTTCTTACAATCAAAATCTCGCCAATGGCAGTTATGATTTAGCTAACAGAGACTTTTGCCAAGGAAATCAGCTAAATTAC
P10.abi            ----------------------------------------------------------------------------------------------------
P12.abi            ----------------------------------------------------------------------------------------------------
```

```
ref/ΔDUF(630-709)  TACAAGCAGGAAATACCCGTGCAACCAACTACCTCATATTCCATTCAGAATTTATACAGTTACGAGAACCAGCCCCAGCCCAGCGATGAATGTACTCTCC
                    Y  K  Q  E  I  P  V  Q  P  T  T  S  Y  S  I  Q  N  L  Y  S  Y  E  N  Q  P  Q  P  S  D  E  C  T  L

SqrP2.abi          ----------------------------------------------------------------------------------------------------
P1.abi             ----------------------------------------------------------------------------------------------------
P3.abi             ----------------------------------------------------------------------------------------------------
P4.abi             ----------------------------------------------------------------------------------------------------
P6.abi             ----------------------------------------------------------------------------------------------------
P8.abi             TACAAGCAGGAAATACCCGTGCAACCAACTACCTCATATTCCATTCAGAATTTATACAGTTACGAGAACCAGCCCCAGCCCAGCGATGAATGTACTCTCC
P10.abi            ----------------------------------------------------------------------------------------------------
P12.abi            ----------------------------------------------------------------------------------------------------
```

```
ref/ΔDUF(630-709) TGAGTAATAAATACCTTGATGGAAATGCTAACAAATCTACCTCAGATGGAAGTCCTGTGATGGCCGTAATGCCTGGTACGACAGACACTATTCAAGTGCT
                  L  S  N  K  Y  L  D  G  N  A  N  K  S  T  S  D  G  S  P  V  M  A  V  M  P  G  T  T  D  T  I  Q  V  L

SqrP2.abi         ----------------------------------------------------------------------------------------------------
P1.abi            ----------------------------------------------------------------------------------------------------
P3.abi            ----------------------------------------------------------------------------------------------------
P4.abi            ----------------------------------------------------------------------------------------------------
P6.abi            ----------------------------------------------------------------------------------------------------
P8.abi            TGAGTAATAAATACCTTGATGGAAATGCTAACAAATCTACCTCAGATGGAAGTCCTGTGATGGCCGTAATGCCTGGTACGACAGACACTATTCAAGTGCT
P10.abi           ----------------------------------------------------------------------------------------------------
P12.abi           ----------------------------------------------------------------------------------------------------
```

```
                                                                                                 Tth111I
ref/ΔDUF(630-709) CAAGGGCAGGATGGATTCTGAGCAGAGCCCTTCTATTGGGTACTCCTCAAGGACTCTTGGCCCCAATCCTGGACTTATTCTTCAGGCTTTGACTCTGTCA
                    K  G  R  M  D  S  E  Q  S  P  S  I  G  Y  S  S  R  T  L  G  P  N  P  G  L  I  L  Q  A  L  T  L  S

SqrP2.abi         ----------------------------------------------------------------------------------------------------
P1.abi            ----------------------------------------------------------------------------------------------------
P3.abi            ----------------------------------------------------------------------------------------------------
P4.abi            ----------------------------------------------------------------------------------------------------
P6.abi            ----------------------------------------------------------------------------------------------------
P8.abi            CAAGGGCAGGATGGATTCTGAGCAGAGCCCTTCTATTGGGTACTCCTCAAGGACTCTTGGCCCCAATCCTGGACTTATTCTTCAGGCTTTGACTCTGTCA
P10.abi           ----------------------------------------------------------------------------------------------------
P12.abi           ----------------------------------------------------------------------------------------------------
```

```
                                                                                    DraI       SphI
ref/ΔDUF(630-709) AACGCTAGTGATGGATTTAACCTGGAGCGGCTTGAAATGCTTGGCGACTCCTTTTTAAAGCATGCCATCACCACATATCTATTTTGCACTTACCCTGATG
                   N  A  S  D  G  F  N  L  E  R  L  E  M  L  G  D  S  F  L  K  H  A  I  T  T  Y  L  F  C  T  Y  P  D

SqrP2.abi         ----------------------------------------------------------------------------------------------------
P1.abi            ----------------------------------------------------------------------------------------------------
P3.abi            ----------------------------------------------------------------------------------------------------
P4.abi            ----------------------------------------------------------------------------------------------------
P6.abi            ----------------------------------------------------------------------------------------------------
P8.abi            AACGCTAGTGATGGATTTAACCTGGAGCGGCTTGAAATGCTTGGCGACTCCTTTTTAAAGCATGCCATCACCACATATCTATTTTGCACTTACCCTGATG
P10.abi           ----------------------------------------------------------------------------------------------------
P12.abi           ----------------------------------------------------------------------------------------------------
```

```
                                                                                                          BstXI
ref/ΔDUF(630-709) CGCATGAGGGCCGCCTTTCATATATGAGAAGCAAAAAGGTCAGCAACTGTAATCTGTATCGCCTTGGAAAAAAGAAGGGACTACCCAGCCGCATGGTGGT
                  A  H  E  G  R  L  S  Y  M  R  S  K  K  V  S  N  C  N  L  Y  R  L  G  K  K  K  G  L  P  S  R  M  V  V

SqrP2.abi         ----------------------------------------------------------------------------------------------------
P1.abi            ----------------------------------------------------------------------------------------------------
P3.abi            ----------------------------------------------------------------------------------------------------
P4.abi            ----------------------------------------------------------------------------------------------------
P6.abi            ----------------------------------------------------------------------------------------------------
P8.abi            CGCATGAGGGCCGCCTTTCATATATGAGAAGCAAAAAGGTCAGCAACTGTAATCTGTATCGCCTTGGAAAAAAGAAGGGACTACCCAGCCGCATGGTGGT
P10.abi           ----------------------------------------------------------------------------------------------------
P12.abi           ----------------------------------------------------------------------------------------------------
```

```
                  SspI
ref/ΔDUF(630-709) GTCAATATTTGATCCCCCTGTGAATTGGCTTCCTCCTGGTTATGTAGTAAATCAAGACAAAAGCAACACAGATAAATGGGAAAAAGATGAAATGACAAAA
                   S  I  F  D  P  P  V  N  W  L  P  P  G  Y  V  V  N  Q  D  K  S  N  T  D  K  W  E  K  D  E  M  T  K

SqrP2.abi         ----------------------------------------------------------------------------------------------------
P1.abi            ----------------------------------------------------------------------------------------------------
P3.abi            ----------------------------------------------------------------------------------------------------
P4.abi            ----------------------------------------------------------------------------------------------------
P6.abi            ----------------------------------------------------------------------------------------------------
P8.abi            GTCAATATTTGATCCCCCTGTGAATTGGCTTCCTCCTGGTTATGTAGTAAATCAAGACAAAAGCAACACAGATAAATGGGAAAAAGATGAAATGACAAAA
P10.abi           ----------------------------------------------------------------------------------------------------
P12.abi           ----------------------------------------------------------------------------------------------------
```

```
                      SphI
ref/ΔDUF(630-709) GACTGCATGCTGGCGAATGGCAAACTGGATGAGGATTACGAGGAGGAGGATGAGGAGGAGGAGAGCCTGATGTGGAGGGCTCCGAAGGAAGAGGCTGACT
                  D  C  M  L  A  N  G  K  L  D  E  D  Y  E  E  E  D  E  E  E  E  S  L  M  W  R  A  P  K  E  E  A  D

SqrP2.abi         ----------------------------------------------------------------------------------------------------
P1.abi            ----------------------------------------------------------------------------------------------------
P3.abi            ----------------------------------------------------------------------------------------------------
P4.abi            ----------------------------------------------------------------------------------------------------
P6.abi            ----------------------------------------------------------------------------------------------------
P8.abi            GACTGCATGCTGGCGAATGGCAAACTGGATGAGGATTACGAGGAGGAGGATGAGGAGGAGGAGAGCCTGATGTGGAGGGCTCCGAAGGAAGAGGCTGACT
P10.abi           --------------------------GGATGAGGATTACGAGGAGGAGGATGAGGAGGAGGAGAGCCTGATGTGGAGGGCTCCGAAGGAAGAGGCTGACT
P12.abi           ----------------------------------------------------------------------------------------------------
```

```
                  BclI*
ref/ΔDUF(630-709) ATGAAGATGATTTCCTGGAGTATGATCAGGAACATATCAGATTTATAGATAATATGTTAATGGGGTCAGGAGCTTTTGTAAAGAAAATCTCTCTTTCTCC
                  Y  E  D  D  F  L  E  Y  D  Q  E  H  I  R  F  I  D  N  M  L  M  G  S  G  A  F  V  K  K  I  S  L  S  P

SqrP2.abi         ----------------------------------------------------------------------------------------------------
P1.abi            ----------------------------------------------------------------------------------------------------
P3.abi            ----------------------------------------------------------------------------------------------------
P4.abi            ----------------------------------------------------------------------------------------------------
P6.abi            ----------------------------------------------------------------------------------------------------
P8.abi            ATGAAGATGATTTCCTGGAGTATGATCAGGAACATATCAGATTTATAGATAATATGTTAAT---------------------------------------
P10.abi           ATGAAGATGATTTCCTGGAGTATGATCAGGAACATATCAGATTTATAGATAATATGTTAATGGGGTCAGGAGCTTTTGTAAAGAAAATCTCTCTTTCTCC
P12.abi           ----------------------------------------------------------------------------------------------------
```

```
                  NdeI
ref/ΔDUF(630-709) TTTTTCAACCACTGATTCTGCATATGAATGGAAAATGCCCAAAAAATCCTCCTTAGGTAGTATGCCATTTTCATCAGATTTTGAGGATTTTGACTACAGC
                    F  S  T  T  D  S  A  Y  E  W  K  M  P  K  K  S  S  L  G  S  M  P  F  S  S  D  F  E  D  F  D  Y  S

SqrP2.abi         ----------------------------------------------------------------------------------------------------
P1.abi            ----------------------------------------------------------------------------------------------------
P3.abi            ----------------------------------------------------------------------------------------------------
P4.abi            ----------------------------------------------------------------------------------------------------
P6.abi            ----------------------------------------------------------------------------------------------------
P8.abi            ----------------------------------------------------------------------------------------------------
P10.abi           TTTTTCAACCACTGATTCTGCATATGAATGGAAAATGCCCAAAAAATCCTCCTTAGGTAGTATGCCATTTTCATCAGATTTTGAGGATTTTGACTACAGC
P12.abi           ----------------------------------------------------------------------------------------------------
```

```
                                   BamHI
ref/ΔDUF(630-709) TCTTGGGATGCAATGTGCTATCTGGATCCTAGCAAAGCTGTTGAAGAAGATGACTTTGTGGTGGGGTTCTGGAATCCATCAGAAGAAAACTGTGGTGTTG
                   S  W  D  A  M  C  Y  L  D  P  S  K  A  V  E  E  D  D  F  V  V  G  F  W  N  P  S  E  E  N  C  G  V

SqrP2.abi         ----------------------------------------------------------------------------------------------------
P1.abi            ----------------------------------------------------------------------------------------------------
P3.abi            ----------------------------------------------------------------------------------------------------
P4.abi            ----------------------------------------------------------------------------------------------------
P6.abi            ----------------------------------------------------------------------------------------------------
P8.abi            ----------------------------------------------------------------------------------------------------
P10.abi           TCTTGGGATGCAATGTGCTATCTGGATCCTAGCAAAGCTGTTGAAGAAGATGACTTTGTGGTGGGGTTCTGGAATCCATCAGAAGAAAACTGTGGTGTTG
P12.abi           ----------------------------------------------------------------------------------------------------
```

```
ref/ΔDUF(630-709) ACACGGGAAAGCAGTCCATTTCTTACGACTTGCACACTGAGCAGTGTATTGCTGACAAAAGCATAGCGGACTGTGTGGAAGCCCTGCTGGGCTGCTATTT
                  D  T  G  K  Q  S  I  S  Y  D  L  H  T  E  Q  C  I  A  D  K  S  I  A  D  C  V  E  A  L  L  G  C  Y  L

SqrP2.abi         ----------------------------------------------------------------------------------------------------
P1.abi            ----------------------------------------------------------------------------------------------------
P3.abi            ----------------------------------------------------------------------------------------------------
P4.abi            ----------------------------------------------------------------------------------------------------
P6.abi            ----------------------------------------------------------------------------------------------------
P8.abi            ----------------------------------------------------------------------------------------------------
P10.abi           ACACGGGAAAGCAGTCCATTTCTTACGACTTGCACACTGAGCAGTGTATTGCTGACAAAAGCATAGCGGACTGTGTGGAAGCCCTGCTGGGCTGCTATTT
P12.abi           ----------------------------------------------------------------------------------------------------
```

```
                      PvuII
ref/ΔDUF(630-709)  AACCAGCTGTGGGGAGAGGGCTGCTCAGCTTTTCCTCTGTTCACTGGGGCTGAAGGTGCTCCCGGTAATTAAAAGGACTGATCGGGAAAAGGCCCTGTGC
                    T  S  C  G  E  R  A  A  Q  L  F  L  C  S  L  G  L  K  V  L  P  V  I  K  R  T  D  R  E  K  A  L  C

SqrP2.abi          ----------------------------------------------------------------------------------------------------
P1.abi             ----------------------------------------------------------------------------------------------------
P3.abi             ----------------------------------------------------------------------------------------------------
P4.abi             ----------------------------------------------------------------------------------------------------
P6.abi             ----------------------------------------------------------------------------------------------------
P8.abi             ----------------------------------------------------------------------------------------------------
P10.abi            AACCAGCTGTGGGGAGAGGGCTGCTCAGCTTTTCCTCTGTTCACTGGGGCTGAAGGTGCTCCCGGTAATTAAAAGGACTGATCGGGAAAAGGCCCTGTGC
P12.abi            ----------------------------------------------------------------------------------------------------
```

```
                                                                                             BalI
ref/ΔDUF(630-709)  CCTACTCGGGAGAATTTCAACAGCCAACAAAAGAACCTTTCAGTGAGCTGTGCTGCTGCTTCTGTGGCCAGTTCACGCTCTTCTGTATTGAAAGACTCGG
                    P  T  R  E  N  F  N  S  Q  Q  K  N  L  S  V  S  C  A  A  A  S  V  A  S  S  R  S  S  V  L  K  D  S

SqrP2.abi          ----------------------------------------------------------------------------------------------------
P1.abi             ----------------------------------------------------------------------------------------------------
P3.abi             ----------------------------------------------------------------------------------------------------
P4.abi             ----------------------------------------------------------------------------------------------------
P6.abi             ----------------------------------------------------------------------------------------------------
P8.abi             ----------------------------------------------------------------------------------------------------
P10.abi            CCTACTCGGGAGAATTTCAACAGCCAACAAAAGAACCTTTCAGTGAGCTGTGCTGCTGCTTCTGTGGCCAGTTCACGCTCTTCTGTATTGAAAGACTCGG
P12.abi            ----------------------------------------------------------------------------------------------------
```

```
                                                                                  BclI*
ref/ΔDUF(630-709) AATATGGTTGTTTGAAGATTCCACCAAGATGTATGTTTGATCATCCAGATGCAGATAAAACACTGAATCACCTTATATCGGGGTTTGAAAATTTTGAAAA
                  E  Y  G  C  L  K  I  P  P  R  C  M  F  D  H  P  D  A  D  K  T  L  N  H  L  I  S  G  F  E  N  F  E  K

SqrP2.abi         ----------------------------------------------------------------------------------------------------
P1.abi            ----------------------------------------------------------------------------------------------------
P3.abi            ----------------------------------------------------------------------------------------------------
P4.abi            ----------------------------------------------------------------------------------------------------
P6.abi            ----------------------------------------------------------------------------------------------------
P8.abi            ----------------------------------------------------------------------------------------------------
P10.abi           AATATGGTTGTTTGAAGATTCCACCAAGATGTATGTTTGATCATCCAGATGCAGATAAAACACTGAATCACCTTATATCGGGGTTTGAAAATTTTGAAAA
P12.abi           ----------------------------------------------------------------------------------------------------
```

```
                                                                                                                  Eco47I
ref/ΔDUF(630-709) GAAAATCAACTACAGATTCAAGAATAAGGCTTACCTTCTCCAGGCTTTTACACATGCCTCCTACCACTACAATACTATCACTGATTGTTACCAGCGCTTA
                    K  I  N  Y  R  F  K  N  K  A  Y  L  L  Q  A  F  T  H  A  S  Y  H  Y  N  T  I  T  D  C  Y  Q  R  L

SqrP2.abi         ----------------------------------------------------------------------------------------------------
P1.abi            ----------------------------------------------------------------------------------------------------
P3.abi            ----------------------------------------------------------------------------------------------------
P4.abi            ----------------------------------------------------------------------------------------------------
P6.abi            ----------------------------------------------------------------------------------------------------
P8.abi            ----------------------------------------------------------------------------------------------------
P10.abi           GAAAATCAACTACAGATTCAAGAATAAGGCTTACCTTCTCCAGGCTTTTACACATGCCTCCTACCACTACAATACTATCACTGATTGTTACCAGCGCTTA
P12.abi           ----------------------------------------------------------------------------------------------------
```

```
                                                                                                                          SmaI
                  II EcoRI                                                                     SacII            XmaI
ref/ΔDUF(630-709) GAATTCCTGGGAGATGCGATTTTGGACTACCTCATAACCAAGCACCTTTATGAAGACCCGCGGCAGCACTCCCCGGGGGTCCTGACAGACCTGCGGTCTG
                   E  F  L  G  D  A  I  L  D  Y  L  I  T  K  H  L  Y  E  D  P  R  Q  H  S  P  G  V  L  T  D  L  R  S

SqrP2.abi         ----------------------------------------------------------------------------------------------------
P1.abi            ----------------------------------------------------------------------------------------------------
P3.abi            ----------------------------------------------------------------------------------------------------
P4.abi            ----------------------------------------------------------------------------------------------------
P6.abi            ----------------------------------------------------------------------------------------------------
P8.abi            ----------------------------------------------------------------------------------------------------
P10.abi           GAATTCCTGGGAGATGCGATTTTGGACTACCTCATAACCAAGCACCTTTATGAAGACCCGCGGCAGCACTCCCCGGGGGTCCTGACAGACCTGCGGTCTG
P12.abi           -------------------------------------------------------------GGCAGCACTCCCCGGGGGTCCTGACAGACCTGCGGTCTG
```

```
                                                                                                                              EcoICRI
                                                                                         ScaI                  Bpu10I SacI
ref/ΔDUF(630-709) CCCTGGTCAACAACACCATCTTTGCATCGCTGGCTGTAAAGTACGACTACCACAAGTACTTCAAAGCTGTCTCTCCTGAGCTCTTCCATGTCATTGATGA
                  A  L  V  N  N  T  I  F  A  S  L  A  V  K  Y  D  Y  H  K  Y  F  K  A  V  S  P  E  L  F  H  V  I  D  D

SqrP2.abi         ----------------------------------------------------------------------------------------------------
P1.abi            ----------------------------------------------------------------------------------------------------
P3.abi            ----------------------------------------------------------------------------------------------------
P4.abi            ----------------------------------------------------------------------------------------------------
P6.abi            ----------------------------------------------------------------------------------------------------
P8.abi            ----------------------------------------------------------------------------------------------------
P10.abi           CCCTGGTCAACAACACCATCTTTGCATCGCTGGCTGTAAAGTACGACTACCACAAGTACTTCAAAGCTGTCTCTCCTGAGCTCTTCCATGTCATTGATGA
P12.abi           CCCTGGTCAACAACACCATCTTTGCATCGCTGGCTGTAAAGTACGACTACCACAAGTACTTCAAAGCTGTCTCTCCTGAGCTCTTCCATGTCATTGATGA
```

```
                    |Bpu10I |BglII
ref/ΔDUF(630-709) CTTTGTGCAGTTTCAGCTTGAGAAGAATGAAATGCAAGGAATGGATTCTGAGCTTAGGAGATCTGAGGAGGATGAAGAGAAAGAAGAGGATATTGAAGTT
                   F  V  Q  F  Q  L  E  K  N  E  M  Q  G  M  D  S  E  L  R  R  S  E  E  D  E  E  K  E  E  D  I  E  V

SqrP2.abi         ----------------------------------------------------------------------------------------------------
P1.abi            ----------------------------------------------------------------------------------------------------
P3.abi            ----------------------------------------------------------------------------------------------------
P4.abi            ----------------------------------------------------------------------------------------------------
P6.abi            ----------------------------------------------------------------------------------------------------
P8.abi            ----------------------------------------------------------------------------------------------------
P10.abi           CTTTGTGCAGTTTCAGCTTGA-------------------------------------------------------------------------------
P12.abi           CTTTGTGCAGTTTCAGCTTGAGAAGAATGAAATGCAAGGAATGGATTCTGAGCTTAGGAGATCTGAGGAGGATGAAGAGAAAGAAGAGGATATTGAAGTT
```

```
                  |NcoI                                     |BstXI
ref/ΔDUF(630-709) CCAAAGGCCATGGGGGATATTTTTGAGTCGCTTGCTGGTGCCATTTACATGGATAGTGGGATGTCACTGGAGACAGTCTGGCAGGTGTACTATCCCATGA
                   P  K  A  M  G  D  I  F  E  S  L  A  G  A  I  Y  M  D  S  G  M  S  L  E  T  V  W  Q  V  Y  Y  P  M

SqrP2.abi         ----------------------------------------------------------------------------------------------------
P1.abi            ----------------------------------------------------------------------------------------------------
P3.abi            ----------------------------------------------------------------------------------------------------
P4.abi            ----------------------------------------------------------------------------------------------------
P6.abi            ----------------------------------------------------------------------------------------------------
P8.abi            ----------------------------------------------------------------------------------------------------
P10.abi           ----------------------------------------------------------------------------------------------------
P12.abi           CCAAAGGCCATGGGGGATATTTTTGAGTCGCTTGCTGGTGCCATTTACATGGATAGTGGGATGTCACTGGAGACAGTCTGGCAGGTGTACTATCCCATGA
```

```
ref/ΔDUF(630-709) TGCGGCCACTAATAGAAAAGTTTTCTGCAAATGTACCCCGTTCCCCTGTGCGAGAATTGCTTGAAATGGAACCAGAAACTGCCAAATTTAGCCCGGCTGA
                  M  R  P  L  I  E  K  F  S  A  N  V  P  R  S  P  V  R  E  L  L  E  M  E  P  E  T  A  K  F  S  P  A  E

SqrP2.abi         ----------------------------------------------------------------------------------------------------
P1.abi            ----------------------------------------------------------------------------------------------------
P3.abi            ----------------------------------------------------------------------------------------------------
P4.abi            ----------------------------------------------------------------------------------------------------
P6.abi            ----------------------------------------------------------------------------------------------------
P8.abi            ----------------------------------------------------------------------------------------------------
P10.abi           ----------------------------------------------------------------------------------------------------
P12.abi           TGCGGCCACTAATAGAAAAGTTTTCTGCAAATGTACCCCGTTCCCCTGTGCGAGAATTGCTTGAAATGGAACCAGAAACTGCCAAATTTAGCCCGGCTGA
```

```
                                                                                          |DraI                 |PstI
ref/ΔDUF(630-709) GAGAACTTACGACGGGAAGGTCAGAGTCACTGTGGAAGTAGTAGGAAAGGGGAAATTTAAAGGTGTTGGTCGAAGTTACAGGATTGCCAAATCTGCAGCA
                    R  T  Y  D  G  K  V  R  V  T  V  E  V  V  G  K  G  K  F  K  G  V  G  R  S  Y  R  I  A  K  S  A  A

SqrP2.abi         ----------------------------------------------------------------------------------------------------
P1.abi            ----------------------------------------------------------------------------------------------------
P3.abi            ----------------------------------------------------------------------------------------------------
P4.abi            ----------------------------------------------------------------------------------------------------
P6.abi            ----------------------------------------------------------------------------------------------------
P8.abi            ----------------------------------------------------------------------------------------------------
P10.abi           ----------------------------------------------------------------------------------------------------
P12.abi           GAGAACTTACGACGGGAAGGTCAGAGTCACTGTGGAAGTAGTAGGAAAGGGGAAATTTAAAGGTGTTGGTCGAAGTTACAGGATTGCCAAATCTGCAGCA
```

```
ref/ΔDUF(630-709) GCAAGAAGAGCCCTCCGAAGCCTCAAAGCTAATCAACCTCAGGTTCCCAATAGCGGTGGCGGAGGTTCTGGAGGCGGTGGAAGTGACTACAAGGACGACG
                   A  R  R  A  L  R  S  L  K  A  N  Q  P  Q  V  P  N  S  G  G  G  G  S  G  G  G  G  S  D  Y  K  D  D

SqrP2.abi         ----------------------------------------------------------------------------------------------------
P1.abi            ----------------------------------------------------------------------------------------------------
P3.abi            ----------------------------------------------------------------------------------------------------
P4.abi            ----------------------------------------------------------------------------------------------------
P6.abi            ----------------------------------------------------------------------------------------------------
P8.abi            ----------------------------------------------------------------------------------------------------
P10.abi           ----------------------------------------------------------------------------------------------------
P12.abi           GCAAGAAGAGCCCTCCGAAGCCTCAAAGCTAATCAACCTCAGGTTCCCAATAGCGGTGGCGGAGGTTCTGGAGGCGGTGGAAGTGACTACAAGGACGACG
```

```
                                                                                                 XhoI
ref/ΔDUF(630-709) ATGACAAGGATTACAAAGACGATGATGACAAGGACTATAAGGACGATGACGATAAGTAACATTTGGTTTAGTGTACAATATCTCCTCGAGCATTTGGTTT
                  D  D  K  D  Y  K  D  D  D  D  K  D  Y  K  D  D  D  D  K  *  H  L  V  *  C  T  I  S  P  R  A  F  G  L
                                                                                  STOP

SqrP2.abi         ----------------------------------------------------------------------------------------------------
P1.abi            ----------------------------------------------------------------------------------------------------
P3.abi            ----------------------------------------------------------------------------------------------------
P4.abi            ----------------------------------------------------------------------------------------------------
P6.abi            ----------------------------------------------------------------------------------------------------
P8.abi            ----------------------------------------------------------------------------------------------------
P10.abi           ----------------------------------------------------------------------------------------------------
P12.abi           ATGACAAGGATTACAAAGACGATGATGACAAGGACTATAAGGACGATGACGATAAGTAACATTTGGTTTAGTGTACAATATCTCCTCGAG----------
```
